# Supplementary material for: piRNAs Can Trigger a Multigenerational Epigenetic Memory in the Germline of C. elegans
Source: Cell. 2012 Jul 6;150(1):88–99. doi: 10.1016/j.cell.2012.06.018 (PMC3464430; doi:10.1016/j.cell.2012.06.018)
Supplement: Table S1. Strains Used in This Study, Related to Experimental Procedures [file mmc1.pdf]

Table S1. Strains Used in This Study, Related to Experimental Procedures

Strain	genotype	comment
CB120	*unc-4(e120) II*	
CB138	*unc-24(e138) IV*	
CB164	*dpy-17(e164) III*	
CB187	*rol-6(e187) II*	
CB450	*unc-13(e450) I*	
DR101	*dpy-5(e61) unc-55(e1170) I*	
DR103	*dpy-10(e128) unc-4(e120) II*	
FX1200	*wago-9(tm1200) III*	
SX461	*mjIs31 II; unc-119(ed3) III*	Hrde sensor
SX1158	*prg-1(n4357) I; mjIs31 II; prg-2(n4358) IV*	
SX1168	*mjIs31 II; hda-4(ok518) X*	
SX1179	*mjIs31 II; amx-1(ok659) III*	
SX1180	*mjIs31 II; lsd-1(vr12) X*	
SX1206	*spr-5(by134) I; mjIs31 II*	
SX1224	*mjIs31 II; mrg-1(qa6200)/qC1 dpy-19(e1259) glp-1(q339)[qIs26] III*	
SX1315	*mjIs145 II; unc-119(ed3) III*	control sensor (GFP)
SX1316	*mjIs144 II; unc-119(ed3) III*	piRNA sensor 21UR-1 (GFP)
SX1328	*mjIs31 II; unc-32(e189) III ; mut-7(pk204) III*	
SX1329	*sago-2(tm894) I; mjIs31 II*	
SX1353	*mjIs31 II; sago-1(tm1195) V*	
SX1442	*mjIs31 nrde-2(mj168) II*	
SX1580	*mjIs144 II; hpl-1(tm1624) X*	
SX1582	*mjIs144 II; hpl-2(tm1489) III*	
SX1584	*mjIs144 II; hpl-2(1489) III; hpl-1(tm1624) X*	
SX1689	*mjIs144 II; nrde-3(tm1116) X*	
SX1801	*prg-1(n4357) I*	
SX1804	*mjIs144 II; nrde-4(mj249) IV*	
SX1866	*mjSi1 II; unc-119(ed3) III*	Ubiquitous H2B-GFP (*dpy-30* promoter)
SX1888	*prg-1(n4357) I; mjIs144 II*	
SX1900	*mjIs144 II; nrde-4(mj259) IV*	
SX1937	*mjIs144 II; wago-9(mj278) III*	
SX2000	*mjIs144 II; wago-9(tm1200) III*	
SX2078	*mjSi22 I; unc-119(ed3) III*	piRNA sensor 21UR-1 (wormCherry)
SX2127	*mjIs31 II; wago-9(tm1200) III*	
SX2131	*mjIs144 II; met-2(4256) III*	
SX2152	*mjIs144 II; rsd-2(pk3307) IV*	
SX2163	*rsd-6(pk3300) I; mjIs144 II*	
SX2171	*mjIs144 II; nrde-1(gg088) III*	
SX2175	*mjIs144 II; set-25(n5021) III*	
SX2239	*mjIs144 II*	
SX2293	*mjIs31 II; set-25(n5021) III*	
SX2294	*mjIs31 II; met-2(n4256) III*	
SX2348	*mjIs144 II; set-2(4589) III*	
SX2349	*mjIs144 II; set-12(n4442) X*	
SX2350	*mjIs144 II; set-9(n4949) IV*	
SX2351	*set-32(ok1457) I; mjIs144 II*	
SX2352	*met-1(n4337) I; mjIs144 II*	
SX2353	*mjIs144 II; set-6(ok2195) X*	
SX2357	*mjSi22 I; nrde-2(gg091) II*	
SX2358	*mjSi22 I; set-11(n4488) II*	

SX2364	*lin-59(n3192) unc-29(e1072) I; mjIs144 II*
YA10	*rsd-2(yp10) IV*
YA11	*rsd-2(yp11) IV*
YA400	*mjIs144 II*
YA401	*prg-1(n4357) I; mjIs144 II*
YA402	*prg-1(n4357) I; mjIs144 II*
YA403	*prg-1(n4357) I; mjIs144 II*
YA404	*prg-1(n4357) I; mjIs144 II*
YA405	*prg-1(n4357) I; mjIs144 II*
YA406	*prg-1(n4357) I; mjIs144 II*
YA407	*prg-1(n4357) I; mjIs144 II*
YA408	*prg-1(n4357) I; mjIs144 II*
YA409	*dpy-10(e128) mjIs144 nrde-2(gg95) II*
YA410	*dpy-10(e128) mjIs144 nrde-2(gg95) II*
YA411	*dpy-10(e128) mjIs144 nrde-2(gg95) II*
YA412	*mjIs144 II; nrde-1(yp4) III*
YA413	*mjIs144 II; nrde-1(yp4) III*
YA414	*mjIs144 II; nrde-1(yp4) III*
YA415	*mjIs144 II; nrde-1(yp5) III*
YA416	*mjIs144 II; nrde-1(yp5) III*
YA417	*mjIs144 II; nrde-1(yp5) III*
YA1172	*rsd-6(pk3300) I*
