# Supplementary material for: piRNAs Can Trigger a Multigenerational Epigenetic Memory in the Germline of C. elegans
Source: Cell. 2012 Jul 6;150(1):88–99. doi: 10.1016/j.cell.2012.06.018 (PMC3464430; doi:10.1016/j.cell.2012.06.018)
Supplement: Document S1. Article plus Supplemental Information [file mmc2.pdf]

Cell

piRNAs Can Trigger a Multigenerational Epigenetic Memory in the Germline of *C. elegans*

Alyson Ashe,[1,2,7] Alexandra Sapetschnig,[1,2,7] Eva-Maria Weick,[1,2,7] Jacinth Mitchell,[3,4,5,7] Marloes P. Bagijn,[1,2] Amy C. Cording,[1,2] Anna-Lisa Doebley,[4] Leonard D. Goldstein,[1,2,8] Nicolas J. Lehrbach,[1,2] Jérémie Le Pen,[1,2] Greta Pintacuda,[1,6] Aisa Sakaguchi,[3,9] Peter Sarkies,[1,2] Shawn Ahmed,[3,4,5,*] and Eric A. Miska[1,2,*]
[1]Wellcome Trust Cancer Research UK Gurdon Institute
[2]Department of Biochemistry
University of Cambridge, Tennis Court Road, Cambridge CB2 1QN, UK
[3]Department of Genetics
[4]Department of Biology
[5]Curriculum in Genetics and Molecular Biology
University of North Carolina, Chapel Hill, NC 27514
[6]Scuola Normale Superiore, Piazza dei Cavalieri, 7, 56126 Pisa, Italy
[7]These authors contributed equally to this work
[8]Present address: Department of Bioinformatics and Computational Biology, Genentech Inc., South San Francisco, CA 94080, USA
[9]Present address: Institute for Molecular and Cellular Regulation, Gunma University, Maebashi, Gunma 371-8512, Japan
*Correspondence: shawn@med.unc.edu (S.A.), eric.miska@gurdon.cam.ac.uk (E.A.M.)
http://dx.doi.org/10.1016/j.cell.2012.06.018

SUMMARY

Transgenerational effects have wide-ranging implications for human health, biological adaptation, and evolution; however, their mechanisms and biology remain poorly understood. Here, we demonstrate that a germline nuclear small RNA/chromatin pathway can maintain stable inheritance for many generations when triggered by a piRNA-dependent foreign RNA response in *C. elegans*. Using forward genetic screens and candidate approaches, we find that a core set of nuclear RNAi and chromatin factors is required for multigenerational inheritance of environmental RNAi and piRNA silencing. These include a germline-specific nuclear Argonaute HRDE1/WAGO-9, a HP1 ortholog HPL-2, and two putative histone methyltransferases, SET-25 and SET-32. piRNAs can trigger highly stable long-term silencing lasting at least 20 generations. Once established, this long-term memory becomes independent of the piRNA trigger but remains dependent on the nuclear RNAi/chromatin pathway. Our data present a multigenerational epigenetic inheritance mechanism induced by piRNAs.

INTRODUCTION

Since August Weismann (1834–1914) formulated the distinction between innate and acquired characteristics at the end of the 19th century, the debate relating to the inheritance of acquired traits has raised many controversies in the scientific community (Weismann, 1891; Bateson, 1919; Haig, 2006). August Weismann himself theoretically rejected this type of hereditability, arguing that, even though environmental stimuli could provoke adaptive responses in the somatic lineage, no evidence suggested that these changes could be communicated to the germline (Weismann, 1891). However, a number of epigenetic phenomena involving RNA, histone modification, or DNA methylation in many organisms have renewed interest in this area (Varmuza, 2003; Haig, 2006; Daxinger and Whitelaw, 2012). Paramutation is a prime example. In this phenomenon, a silenced allele can act in *trans* on a homologous sequence to cause stable and heritable silencing. This newly silenced allele can now itself act in a paramutagenic fashion to silence other alleles. Paramutation has been described in multiple species, and it seems likely that small RNAs play a key role in the process, although the full mechanisms involved still remain unclear (Stam and Mittelsten Scheid, 2005; Chandler, 2010; Suter and Martin, 2010).

C. elegans has emerged as a key model for the analysis of several related pathways that regulate genes via small RNAs. *C. elegans* is well suited to the analysis of multigenerational effects, due to its short generation time (~3 days) and the ease with which they can be maintained under tightly controlled experimental conditions. In eukaryotes, 20–30 nucleotide (nt) RNAs bound to Argonaute (AGO) protein cofactors are the effectors of a number of gene regulation pathways (Carmell et al., 2002). The discovery of the process of RNA interference (RNAi) has been a major milestone (Fire et al., 1998). While 21–22 nt small interfering RNAs (siRNAs) are the small RNA effectors of RNAi, RNAi can be induced by injection of long double-stranded RNA (dsRNA) or by providing dsRNA environmentally in the food of *C. elegans* (Timmons et al., 2001). In both instances, dsRNA is processed by the RNase Dicer to give rise to primary siRNAs. RNAi effects are generally systemic (soma and germline) and

 ©2012 Elsevier Inc.

are observed in the F1 generation, but the latter requires the generation of secondary siRNAs (Grishok et al., 2000; Pak and Fire, 2007; Sijen et al., 2007; Gu et al., 2009). Secondary siRNAs represent the most abundant class of endogenous small RNAs in *C. elegans*, are RNA-dependent RNA polymerase products, have a 5′ triphosphate, and are predominantly 22 nt in length with a 5′ guanosine (22G-RNAs). Secondary siRNA pathways and RNA-dependent RNA polymerases (RdRPs) have not been found in vertebrates or *Drosophila*, but have been found in many other organisms, including nematodes, plants, fungi, and viruses. Secondary siRNA pathways in *C. elegans* are complex, can involve many different AGO proteins, and are only partly understood (Yigit et al., 2006).

Several studies have reported inheritance of environmental RNAi beyond the F1 generation (Fire et al., 1998; Grishok et al., 2000; Vastenhouw et al., 2006; Alcazar et al., 2008; Gu et al., 2012). In one transgenerational paradigm, small RNA inheritance and histone H3K9me3 marks were observed for at least two generations (Gu et al., 2012). In addition, transgenerational inheritance of viral immunity (Rechavi et al., 2011) and longevity (Greer et al., 2011) were recently reported for *C. elegans*. These data suggest that the biological roles of transgenerational inheritance could be diverse but remain largely speculative. In addition, whether this transmission involves transgenerationally transmitted RNAs or modifications of chromatin is still controversial.

Piwi-interacting RNAs (piRNAs) are distinct from siRNAs and have an evolutionarily conserved role in transposon silencing in the germline in many animals, including nematodes (Malone and Hannon, 2009; Bagijn et al., 2012). *C. elegans* encodes two Piwi clade, AGO superfamily proteins, PRG-1 and PRG-2, although PRG-2 has likely little or no function (Batista et al., 2008; Das et al., 2008; Bagijn et al., 2012). *C. elegans* piRNAs are absent in *prg-1* mutant animals, which exhibit fertility defects. PRG-1 and piRNA expression is restricted to the male and female germline (Batista et al., 2008; Das et al., 2008; Bagijn et al., 2012). The piRNAs of *C. elegans* are 21 nucleotides in length with a 5′ uracil (21U-RNAs) (Ruby et al., 2006; Batista et al., 2008; Das et al., 2008; Wang and Reinke, 2008). *C. elegans* piRNAs derive from two broad clusters on chromosome IV (Ruby et al., 2006) and act in *trans* to regulate endogenous targets in the germline (Bagijn et al., 2012).

Here, we report how transgenerational inheritance of environmental RNAi and the piRNA pathway converge on the same germline nuclear RNAi/chromatin pathway. Both nuclear RNAi factors and chromatin regulators are essential for silencing. This pathway can elicit a long-term epigenetic memory for more than 24 generations. Once established, the initial silencing trigger is no longer required.

RESULTS

A Reporter-Based System to Investigate Transgenerational Gene Silencing in *C. elegans*

To genetically dissect multigenerational gene silencing in *C. elegans*, we developed a heritable environmental RNAi paradigm. Taking advantage of the recent advance in technologies to generate single-copy intrachromosomal transgenes in *C. elegans* (Frøkjaer-Jensen et al., 2008, 2012), we generated a reporter transgene expressing a histone-GFP fusion protein in the germline of *C. elegans* (Figure 1A and Figure S1 available online). The use of such a defined artificial locus combines the ability to map small RNA populations, which was previously not possible using multicopy transgenes (Vastenhouw et al., 2006), with a high-throughput, quantifiable approach that was not possible using an endogenous locus (Alcazar et al., 2008). Eliciting environmental RNAi by feeding transgenic animals with bacteria expressing dsRNA corresponding to the GFP mRNA results in gene-specific silencing of this GFP transgene (P0, Figures 1A and 1B), as expected (Timmons et al., 2001). Transfer of these animals to a neutral environment results in a high level of silenced animals in the F1 generation, again as expected (Figure 1B) (Fire et al., 1998; Grishok et al., 2000). Furthermore, silencing of the transgene is maintained for at least four additional generations in a subpopulation of animals. We quantified this phenomenon in thousands of animals for each generation using flow cytometry and found that inheritance of transgene silencing was maintained in more than 60% of animals for at least four generations (Figures 1C and S2). We conclude that we have established a reporter-based paradigm for the investigation of transgenerational inheritance, the "*h*eritable *R*NAi *de*fective," or "Hrde," sensor.

Multigenerational Gene Silencing Is Associated with Continued Small RNA Expression

As the mechanisms of transgenerational inheritance are currently not understood, we first asked whether the Hrde sensor silencing that we observed is due to posttranscriptional regulation of mRNA or (co-)transcriptional gene regulation. Using quantitative RT-PCR, we tested whether Hrde sensor silencing in the F2 generation affected either. We were able to robustly detect both primary transcript (pre-mRNA) and Hrde transgene mRNA. However, mRNA levels were significantly repressed ($p < 0.05$) in silenced animals as compared to nonsilenced animals (Figure 1D). pre-mRNA levels showed a similar trend. These data suggest that posttranscriptional mechanisms of silencing are required in the Hrde paradigm. We postulated that Hrde sensor transcript availability might result in continued small RNA pathway activity in silenced animals. Therefore, we profiled small RNAs using high-throughput sequencing from animals undergoing environmental RNAi (P0), control RNAi (P0), or at the F4 generation after RNAi. Small RNA libraries were prepared using protocols that did not necessitate the presence of a 5′ monophosphate to capture primary and secondary siRNAs. We detected abundant sense and antisense small RNAs during environmental RNAi (P0 generation) (Figure 1E). These small RNAs had a peak length of 21–22 nt and little bias for the 5′-most nucleotide and likely represent Dicer cleavage products (primary siRNAs) (Figure 1F). In contrast, four generations later, only antisense small RNAs remain with the characteristic signature of secondary siRNAs (22 nucleotide length with a 5′ guanosine bias, 22G RNAs). Given that each generation represents at least a hundred-fold dilution in volume (with more than 200 offspring generated by each hermaphrodite), these secondary siRNAs must be generated de novo in each generation. Animals undergoing control RNAi displayed a peak of small RNAs homologous to cloning sequences flanking the GFP minigene. These

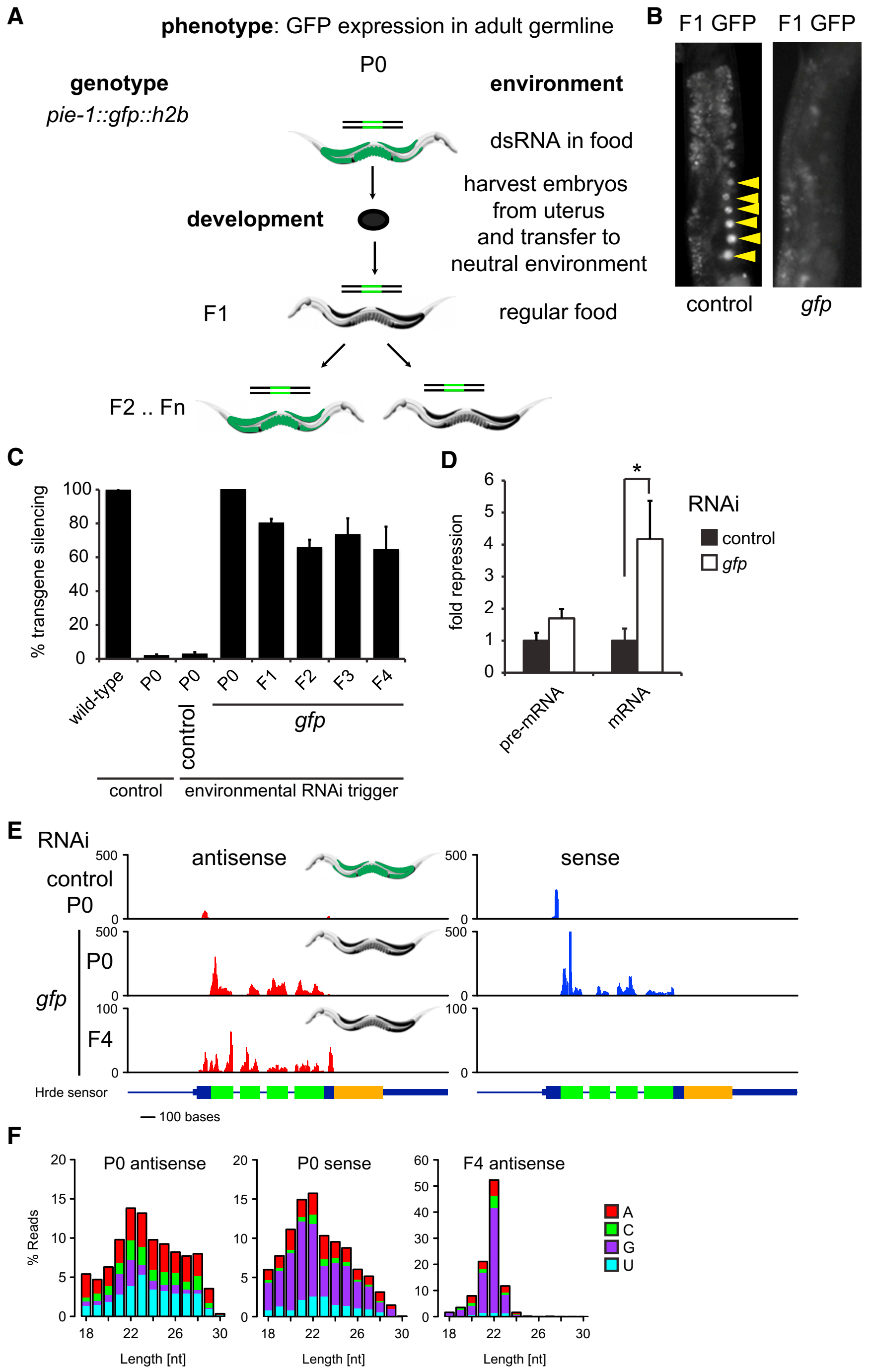

Figure 1. A Novel Inheritance Paradigm Demonstrates that Transgenerational Inheritance Is Associated with Continued Small RNA Production

(A) A diagram of the Hrde sensor inheritance paradigm. Green animals illustrate the germline-expressed GFP sensor, whereas black worms represent silenced animals.

(B) Representative images showing the germline-expressed transgene. The left panel shows the germline of an animal fed control vector, whereas the right panel shows the germline of an animal whose parent was treated with dsRNA targeting the GFP transgene. Arrows show the developing oocytes.

(C) Graph showing the percentage of GFP-silenced animals following exposure to GFP RNAi. Wild-type worms do not contain the hrde sensor; P0-F4 animals carry the sensor and differ only in their exposure to dsRNA. GFP fluorescence of the transgene and the percentage of silenced animals per plate were determined using a large particle biosorter and FlowJo. Ten silenced worms were selected from each plate to produce the next generation. At least 1,000 worms were analyzed per plate with the following number of replicates: P0 (GFP vector) n = 3, F1 n = 18, F2 n = 11, F3 n = 8, F4 n = 5. Error bars represent the SEM. Silencing was normalized to wild-type to account for auto-fluorescence of the intestine.

(D) qRT-PCR showing levels of nascent, unspliced pre-mRNA and mRNA for the GFP transgene in silenced, GFP RNAi treated F2 and control worms. Fold change is shown relative to control and normalized to *ama-1* expression. n = 4, 3, 4, 4 for pre-mRNA control, *gfp*, mRNA control and *gfp*, respectively.

(E) Small RNA reads with unique perfect match in the transgene construct and no perfect match in the reference genome are shown for P0 and F4 L4 stage animals. F4 animals are the offspring of silenced animals in previous generations. Antisense and sense reads are shown in red and blue, respectively. Profiles indicate number of reads per million. Schematic indicates the transgene structure. Blue bars are *pie-1* genomic DNA, 5′ and 3′ UTR, and exon (thin, medium, thick), respectively. Thick green/yellow bars represent GFP/*his-58*, respectively.

(F) Size distribution of small RNA reads in (E). For each size, the relative contribution of small RNAs with a particular 5′ nucleotide is represented in colors as indicated.

Error bars represent SEM. See also Figures S1 and S2.

are homologous to cloning sequences that are present in the RNAi vectors and have no apparent effect on Hrde sensor activity (Figure 1C).

Multigenerational Gene Silencing and piRNA Silencing Depend on Common Nuclear Factors

We recently reported that piRNA-mediated silencing in the *C. elegans* germline results in secondary siRNA-dependent silencing of a “piRNA sensor” and endogenous piRNA targets (Bagijn et al., 2012). Thus, piRNA-mediated silencing might converge on a common downstream multigenerational gene silencing pathway. To this end, we carried out forward genetic screens to identify genes required for either phenomenon using the Hrde and piRNA sensors. Using these two distinct sensors (Figure S1), one silenced by a single endogenous piRNA (piRNA sensor) and the other silenced by heritable environmental RNAi (Hrde sensor), we identified, mapped, and cloned new alleles of three known genes in small RNA pathways: *nrde-2*, *nrde-4*,

Table 1. Multigenerational Environmental RNAi and piRNA Silencing Require a Common Nuclear Pathway

Genotype	Gene Product(s)	Reference Allele Tested	Alleles Described in This Study	Heritable Environmental RNAi Defective	piRNA Silencing Defective
wild-type	NA	NA	NA	–	-
nrde-2	novel	*gg91*, *gg95*	*mj168* (Q135Stp)	+	+[a]
nrde-1	novel	*gg88*, *yp4*, *yp5*		ND	+
nrde-4	novel		*mj249* (Q707Stp) *mj259* (Q663Stp)	ND	+
hrde-1/wago-9	nuclear Argonaute	*tm1200*	*mj278* (P720L)	+	+[b]
nrde-3	nuclear Argonaute	*tm1116*		ND	-[b]
sago-1	Argonaute	*tm1195*		–	–[b]
sago-2	Argonaute	*tm894*		–	–[b]
hpl-2	chromo domain	*tm1489*		ND	+
hpl-1	chromo domain	*tm1624*		ND	–
hpl-1; hpl-2	chromo domains	*tm1489*; *tm1624*		ND	+
set-25	SET domain	*n5021*		+	+
set-32	SET domain	*ok1457*		ND	+
met-2	SET domain	*n4256*		-	–
met-1	SET domain	*n4337*		ND	–
lin-59	SET domain	*n3192*		ND	–
set-2	SET domain	*n4589*		ND	–
set-6	SET domain	*ok2195*		ND	–
set-9	SET domain	*n4949*		ND	–
set-11	SET domain	*n4488*		ND	–[a]
set-12	SET domain	*n4442*		ND	–
hda-4	histone deacetylase	*ok518*		–	ND
mrg-1	chromo domain	*qa6200*		–	ND
spr-5	histone demethylase	*by134*		–	ND
lsd-1	histone demethylase	*vr12*		–	ND
amx-1	amine oxidase	*ok659*		–	ND
prg-1; prg-2	Piwi	*n4357*, *n4358*		–	+[b]
prg-1	Piwi	*n4357*		ND	+[b,d]
rsd-2	novel	*pk3307*		ND	–
rsd-6	Tudor domain	*pk3300*		ND	–
mut-7	RNase D	*pk204*		+[c]	+[b]

For heritable environmental RNAi and piRNA silencing assays, intrachromosomal single-copy transgenes were used as reporters. For heritable environmental RNAi, animals were scored at the F2 generation (see Figure 1A). For piRNA silencing, a sensor for the endogenous piRNA 21UR-1, the "piRNA sensor," on chromosome II was used (Bagijn et al., 2012). ND, not done.

[a]Tested using the piRNA cherry sensor on chromosome I, as described in Bagijn et al., 2012.

[b]Previously reported in Bagijn et al., 2012.

[c]These mutants were already defective in silencing in the F1 generation.

[d]Results are dependent on multigenerational ancestry of the animals (this study).

and *hrde-1/wago-9* (Table 1). Next, combining forward genetic screens with a candidate gene approach, we were surprised to identify a total of eight small RNA or chromatin pathway genes to be required (Table 1). For example, the Hrde sensor was desilenced in *nrde-2*, *hrde-1/wago-9*, and *set-25* mutants (Figure 2A and Table 1). The products of all of these genes are either known to be, or are predicted to be, nuclear. NRDE-2 is a conserved protein involved in nuclear RNAi that is expressed in the nucleus (Guang et al., 2010); SET-25 is a putative histone H3 lysine-9 methyltransferase with a C-terminal SET domain. To our knowledge, this is the first time that a histone-modifying enzyme has been identified as required for multigenerational inheritance. HRDE-1/WAGO-9 is an Argonaute protein. Using immunostaining, we show that it is expressed in the germline (Figure 2B), where it localizes to the nucleus (Figures 2C and 2D). NRDE-2 was recently shown to be important in a similar inheritance paradigm (Gu et al., 2012). However, some genes that were previously reported to be involved in transgenerational effects appeared not to be required for our transgenerational inheritance paradigm, including *hda-4*, *mrg-1* (Vastenhouw et al., 2006) or *spr-5*, *lsd-1*, and *amx-1* (Katz et al., 2009) (Table 1). For the piRNA sensor, aside from proteins that were defined in Hrde

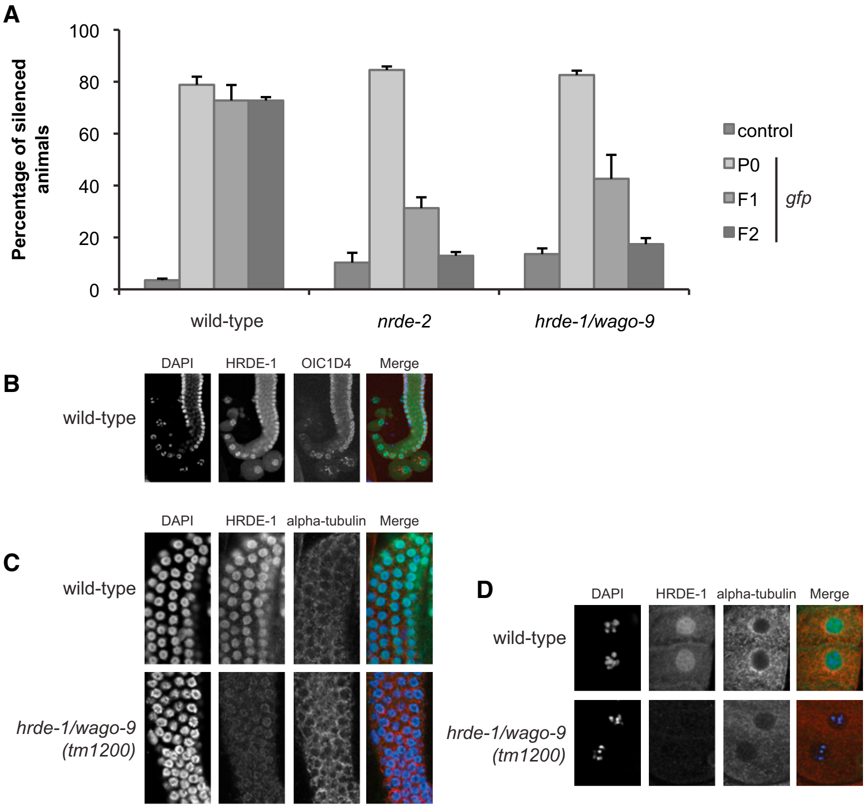

Figure 2. Transgenerational Inheritance Requires NRDE-2 and the Germline-Specific Nuclear Argonaute HRDE-1/WAGO-9

(A) Biosorter analysis of WT, *nrde-2*, and *hrde-1/wago-9* animals showing the failure of heritable silencing in these mutant strains. GFP fluorescence of the transgene and the percentage of silenced animals per plate were determined using a large particle biosorter and FlowJo. Ten silenced worms were selected where possible from each plate to produce the next generation. At least 500 worms were analyzed per plate with the following number of replicates (empty vector, GFP RNAi, F1, F2, respectively). WT, n = 7, 6, 4, 4; *nrde-2*, n = 3, 3, 3, 3; *hrde-1/wago-9*, n = 12, 6, 6, 6. Error bars represent the SEM.

(B) HRDE-/WAGO-9 is expressed in the germline. Wild-type dissected germlines (adults) were stained with anti-HRDE-1/WAGO-9 (green) and a P-granule-specific antibody (OIC1D4, red). DNA was costained with DAPI (blue). Images on the right are merged from all three channels.

(C and D) HRDE-/WAGO-9 is a nuclear protein. Immunostainings were performed on dissected gonads from adult wild-type (N2) or *hrde-1/wago-9 (tm1200)* animals using anti-HRDE-1/WAGO-9 (green) and anti-α-tubulin antibodies (red). DNA was costained with DAPI (blue). Images on the right are merged from all three channels. Images shown are germ cells in the transition zone/pachytene region (C) and oocytes (D).

screens such as NRDE-2, HRDE-1/WAGO-9, and SET-25, we also identified additional nuclear small RNA components and chromatin factors, including NRDE-1, NRDE-4, SET-32, and one of the *C. elegans* heterochromatin protein 1 (HP1) orthologs, HPL-2 (Table 1 and Figure S3). We conclude that there exists a common and specific nuclear RNAi/chromatin pathway in the germline that is required for environmentally induced heritable RNAi- and piRNA-induced silencing.

The Nuclear RNAi/Chromatin Silencing Pathway Acts Downstream of Small RNA Expression in Gene Silencing

To establish a hierarchy in the silencing pathways described here, we asked whether nuclear RNAi/chromatin components are upstream or downstream of secondary siRNA expression and/or stability. First, we analyzed small RNA expression in the Hrde sensor paradigm in wild-type and a *nrde-2* mutant background. In both cases, we find abundant 22G secondary siRNAs that map to the Hrde sensor (Figure 3A). Thus, NRDE-2 is not required for secondary siRNA generation. Next, we tested a chromatin factor using the piRNA sensor. The HP-1 ortholog HPL-2, but not HPL-1, is required for silencing of the piRNA sensor (Table 1 and Figure 3B). Therefore, we asked whether secondary siRNAs are expressed and stable in *hpl-2* mutant animals. Using northern blotting, we show that the piRNA 21UR-1 and a piRNA-sensor-specific 22G RNA (siR22G-1) are dependent on the Piwi protein PRG-1 (Figure 3C). However, both RNAs are present in *hpl-2* and *hpl-2*; *hpl-1* mutant backgrounds, although possibly at reduced levels for siR22G-1. These observations are in agreement with similar observations made for siRNAs in *S. pombe* lacking Swi6/HP1 (Bühler et al., 2006). In addition, we analyzed endogenous targets of the piRNA pathway that we recently identified (Bagijn et al., 2012). We generated small RNA libraries from wild-type and *prg-1* or *hpl-2* mutant animals. 22G secondary siRNAs at endogenous piRNA targets *bath-45* and *zfp-1* are dependent on PRG-1, but not HPL-2 (Figures 3D and 3E). Again, we observed some reduction in RNA levels, consistent with a positive interaction between nuclear RNAi and chromatin regulation. We conclude that the nuclear RNAi/chromatin pathway described here is not essential for secondary siRNA expression or stability.

Multigenerational Gene Silencing and piRNA Silencing Does Not Spread into the Soma

As the nuclear RNAi/chromatin pathway that we describe here utilizes small RNAs, it might act in *trans* on transcripts that share significant sequence similarity. Indeed, using the piRNA sensor, we were able to test this directly. The piRNA sensor is under the transcriptional control of a germline-specific promoter (*mex*-5). Silencing of the piRNA sensor is established through an endogenous piRNA (21-UR-1) with perfect complementarity to a corresponding sequence in the piRNA sensor (Bagijn et al., 2012). A cross of the piRNA sensor strain to a different transgenic strain with a ubiquitously expressed GFP transgene that is not regulated by piRNAs (Figure 4A) results in dominant silencing of both transgenes in the germline of heterozygous F1 animals (Figure 4B), likely via a process termed transitive RNAi (Alder

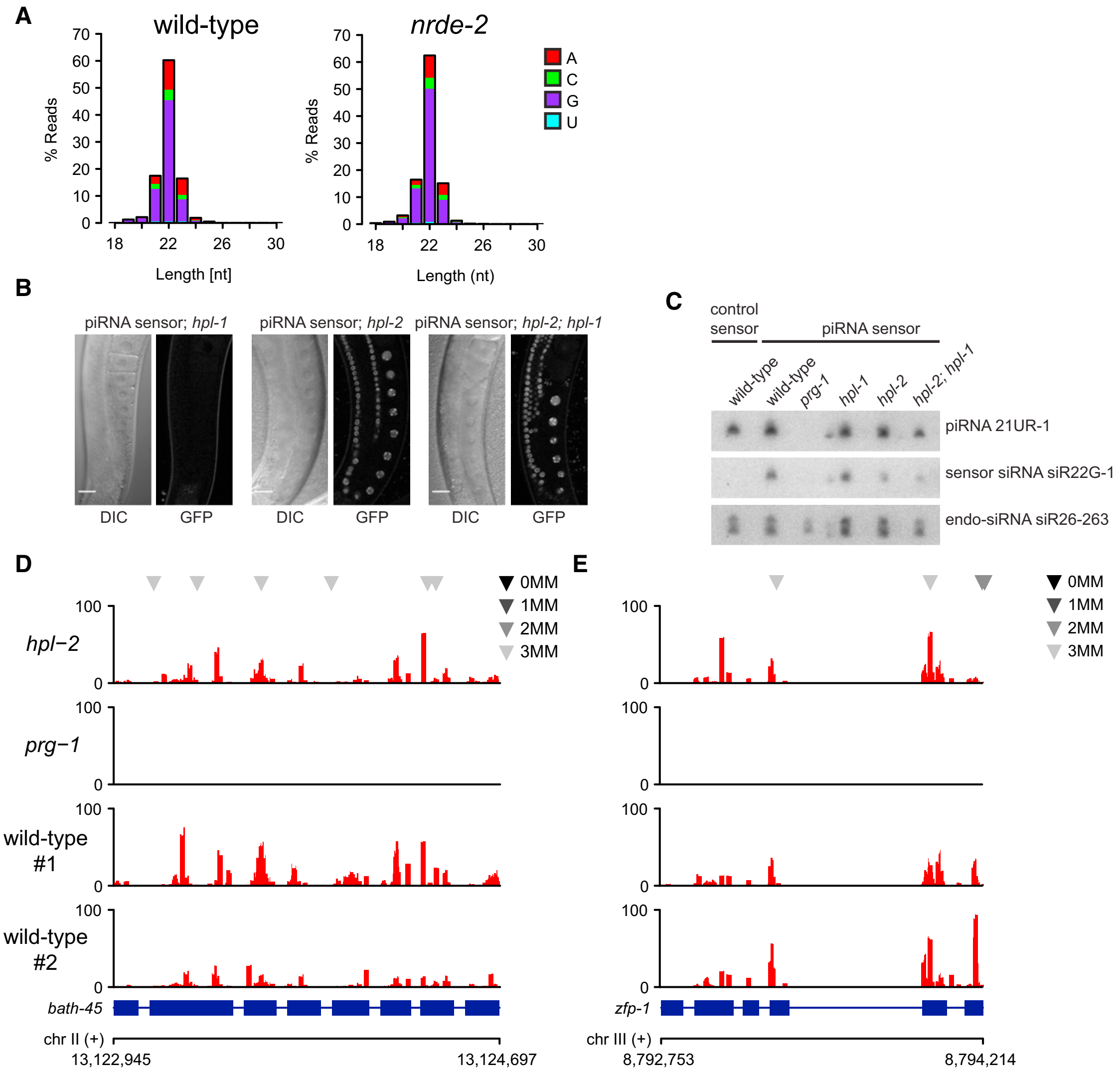

Figure 3. The Germline Nuclear RNAi/Chromatin Pathway Acts Downstream of Small RNA Production and Stability

(A) Size distribution of small RNA reads with unique perfect match in the transgene construct and no perfect match in the reference genome are shown for wild-type and *nrde-2* F2 animals. For each size, the relative contribution of small RNAs with a particular 5′ nucleotide is represented in colors as indicated.

(B) The heterochromatin protein HPL-2 is required for piRNA sensor silencing. DIC and fluorescence microscopy of piRNA sensor germlines in indicated mutant genotypes. Scale bars, 20 μm.

(C) HPL-2 acts downstream of 22G-RNA biogenesis. Northern blot of total RNA from control sensor and indicated piRNA sensor strains. Probes were against piRNA 21UR-1, a piRNA sensor-specific 22G-RNA, and the Piwi-independent endo-siRNA siR26-263. For oligonucleotide sequences, see Bagijn et al., 2012.

(D and E) Antisense 22G-RNA profiles are shown for selected elements. Profiles indicate number of reads per million. piRNA target sites are indicated above each profile as explained in the color key.

See also Figure S3.

et al., 2003). Thus, the nuclear RNAi/chromatin pathway can silence in *trans*. We postulate that this effect is mediated via secondary siRNAs. As exogenous and endogenous RNAi are systemic in *C. elegans* (Fire et al., 1998; Winston et al., 2002), we therefore wondered whether the germline nuclear RNAi/chromatin silencing pathway that we describe here could transcend the germline/soma boundary. We do not find this to be the case, as GFP expression in the *trans*-heterozygous animals (*dpy-30::his-58::gfp::tbb-2*/piRNA sensor) described above remains unaffected in the soma (Figure 4B). We made the same observation using the Hrde-1 sensor and another somatic transgene in an analogous experiment (data not shown). We conclude that, though the nuclear RNAi/chromatin pathway that we describe here can be vertically transmitted, it does not trigger systemic RNAi. This is consistent with recent work demonstrating that secondary siRNAs are not systemically transmitted in the soma of *C. elegans* (Jose et al., 2011). We note that results based on multicopy transgenes that possibly involve dsRNA intermediates could be different from those reported here (Jose et al., 2011).

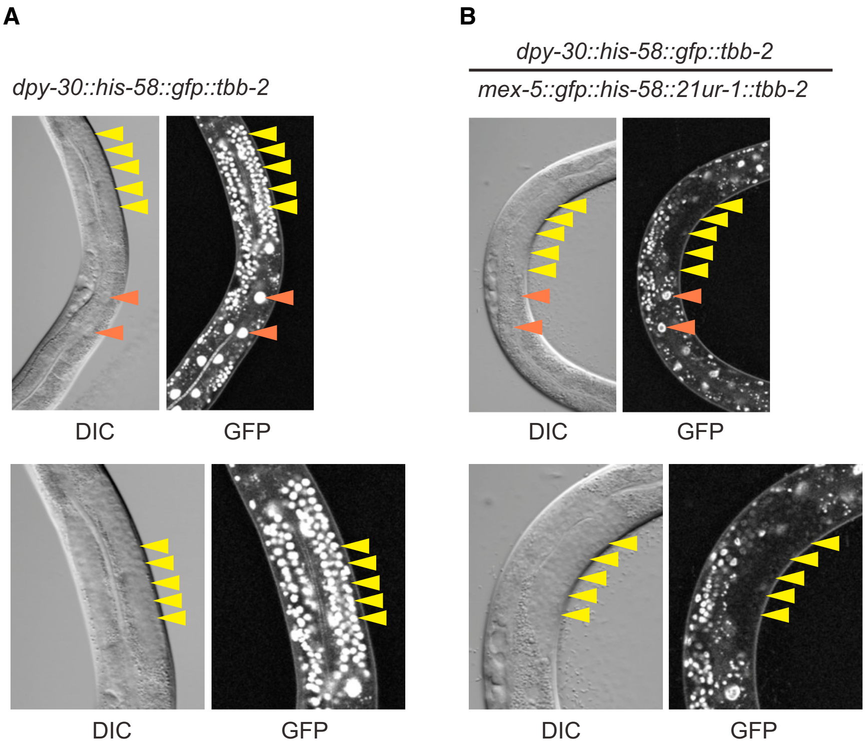

Figure 4. The Germline Nuclear RNAi/Chromatin Pathway Acts in *trans* but Cannot Exit the Germline

(A and B) *trans*-heterozygous animals were generated by crossing SX1866 hermaphrodites with piRNA sensor males. Strain SX1866 expressing H2B-GFP under control of the ubiquitous *dpy-30* promoter was generated by MosSCI into *ttTi5606* on chromosome II (*mjSi1[dpy-30::his-58::gfp::tbb-2]*). DIC and fluorescence microscopy of animals from the parental line (A) or of *trans*-heterozygous animals from the cross (B). Note that the parental line expresses H2B-GFP from two copies in the genome and is therefore brighter. Yellow arrowheads indicate germ cell nuclei; red arrowheads indicate somatic (intestinal) cell nuclei.

piRNAs Can Trigger Long-Term Multigenerational Gene Silencing

Our data demonstrate that environmentally induced multigenerational gene silencing and piRNA silencing converge on a common germline silencing pathway. Can a piRNA therefore trigger multigenerational gene silencing? To address this question, we carried out genetic crosses in which we removed PRG-1 and thereby piRNA function from the piRNA sensor strain (Das et al., 2008; Bagijn et al., 2012). In these circumstances, the piRNA trigger is removed but silencing might be maintained. In a cross of animals homozygous for the piRNA sensor (GFP silenced) with an animal homozygous for the piRNA sensor but in a *prg-1* mutant background (GFP expressed), we generated F1 animals homozygous for the piRNA sensor but heterozygous for the recessive mutation in *prg-1* (Figure S4). Such animals are GFP silenced for several generations, as expected. These heterozygous animals segregate progeny that are homozygous, heterozygous, or wild-type with respect to *prg-1*. We observed piRNA sensor reactivation in *prg-1* homozygous mutants or their immediate offspring. Because all piRNAs are eliminated in *prg-1* mutants (Batista et al., 2008; Das et al., 2008), these data suggested that a piRNA trigger may be required to maintain multigenerational silencing memory.

Next, we recreated a piRNA sensor strain that was mutant for *prg-1* by outcrossing the piRNA sensor and then performing several crosses using mutations that cause visible phenotypes to mark the positions of *prg-1* or the piRNA sensor transgene (see Experimental Procedures). Unexpectedly, 11 *prg-1*; piRNA sensor strains failed to reactivate the piRNA sensor (n = 8 or 3 independent strains created per trial for 2 trials) (Figures 5A and 5B). GFP expression of these *prg-1;* piRNA sensor strains failed to materialize even though many successive generations were scored, which were last analyzed at F16, F17 (three strains), F20, and F24 (six strains) generations. We also observed that silencing can become PRG-1 independent using a second piRNA sensor construct integrated on a different chromosome (the piRNA mCherry sensor; Figure S5). We conclude that germline silencing can persist for many generations even in the absence of a piRNA trigger. It is of interest to note that all crosses that led to trigger-independent maintenance of silencing involved the piRNA sensor transgene being heterozygous for 3–5 generations due to outcrossing.

In contrast to multigenerational silencing of piRNA sensor transgenes in the absence of *prg-1*, mutation of *nrde-1* (*yp4* or *yp5*) or mutation of *nrde-2* (*gg95*) triggered reactivation of outcrossed piRNA sensors (n = 3, 2, and 3, respectively, independently isolated F3 or F4 strains scored) (Figure 5C). All *nrde-2* mutant lines expressed bright GFP from F3 onward. Of five piRNA sensor*; nrde-1* lines, three lines expressed weak GFP signal in all germ cells in the F3 generation, whereas germ cells of all animals scored in piRNA sensor*; nrde-1* lines were uniformly positive for a weak GFP signal by the F4 generation. We conclude that nuclear small RNA factors are required to maintain the silenced state over many generations, whereas the piRNA trigger that initiates silencing becomes dispensable if the silent locus is outcrossed multiple times.

A Tudor domain protein RSD-6 and a novel protein RSD-2 have previously been shown to be required for RNAi responses to environmental dsRNA triggers that target genes expressed in the germline and are proficient for RNAi to some somatic targets, possibly due to dose-dependent RNAi defects (Tijsterman et al., 2004; Merritt et al., 2008; Zhang et al., 2012). The strong germline RNAi defects of *rsd-6* or *rsd-2* suggested that they could function to promote systemic spreading of RNAi from soma to germline (Tijsterman et al., 2004). To determine where *rsd-6* functions to promote germline RNAi, single-copy

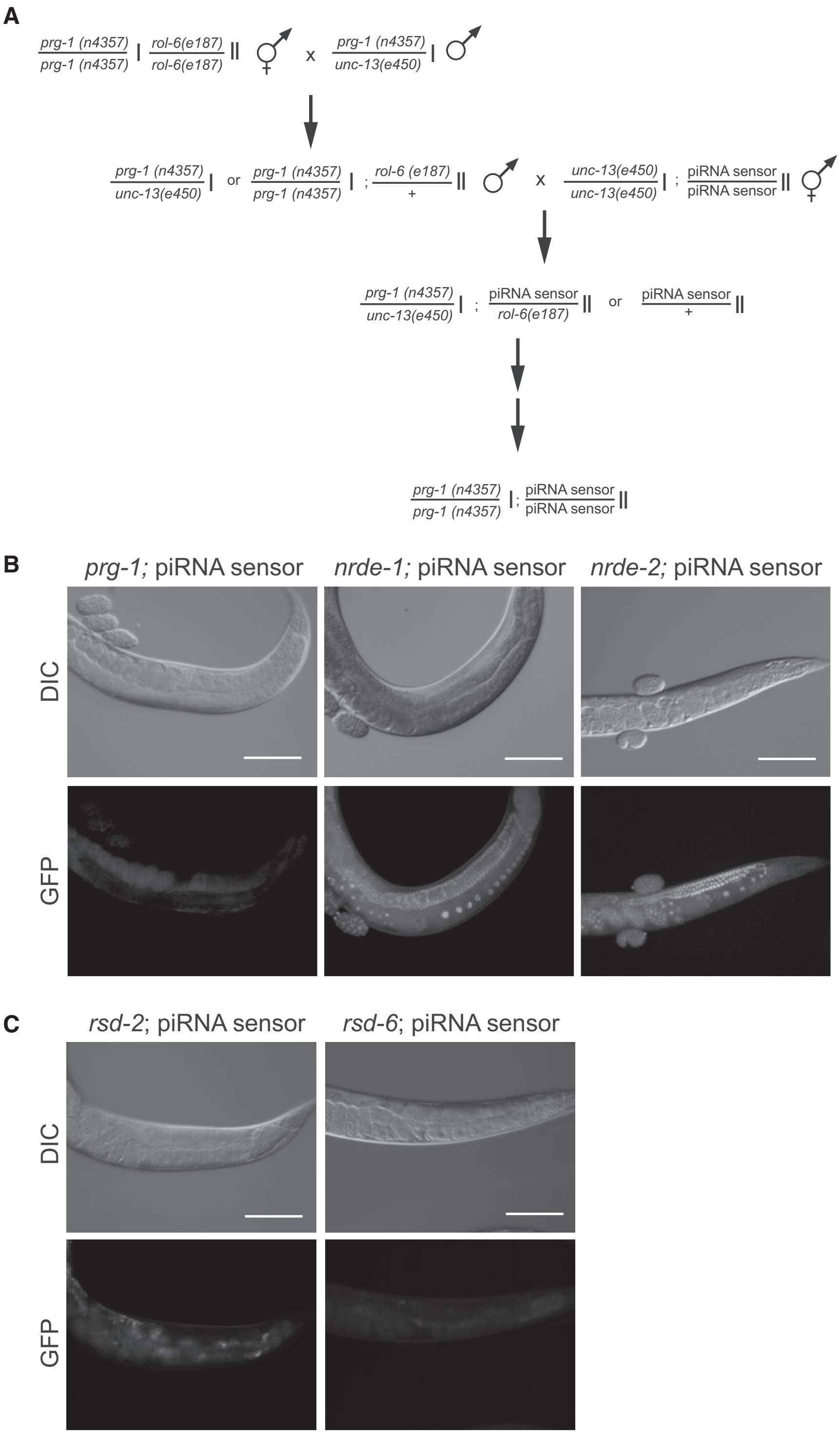

Figure 5. piRNAs Can Induce Stable Multigenerational Inheritance that Does Not Require PRG-1 for Maintenance

(A) Schematic showing generation of *prg-1*; piRNA sensor strain, which has lost the requirement for PRG-1 to maintain transgene silencing (for details of previous outcrosses, see Experimental Procedures). Analogous crosses were performed for nuclear RNAi factors, with nrde-2 requiring further intermediate steps.

(B) Following a number of crosses in a *prg-1*-sufficient background, the piRNA reporter is desilenced in *nrde-1* and *nrde-2*, but not *prg-1*, mutant backgrounds. Differential interference contrast or GFP epifluorescence photos are shown. White bars correspond to 100 μm.

(C) Outcrossed piRNA sensors fail to express GFP in *rsd-2* or *rsd-6* mutant backgrounds. Higher autofluorescence is observed for these strains, which were raised at 25°C, than for those in (B), which were raised at 20°C.

See also Figures S4, S5, and S6.

rsd-6 transgenes driven by the germline-specific *pgl-3* promoter or by the ubiquitous promoter *dpy-30* were created (Frøkjaer-Jensen et al., 2008; Han et al., 2008). Both transgenes rescued an *rsd-6* mutant for the response to dsRNAs targeting the germline-expressed genes *pop-1* or *par-6* (Figure S6), indicating that RSD-6 functions in a cell-autonomous manner within the germline. We created *rsd-6*; piRNA sensor and piRNA sensor; *rsd-2* strains using outcrossed sensor transgenes and observed that these strains were GFP negative when initially created and for many generations thereafter (Figure 5C). These results suggest that piRNA sensor silencing may not depend on systemic RNAi effects (possibly mediated by expression of dsRNA in somatic cells). They also suggest that the response to dsRNA generated in germ cells is unlikely to promote sensor silencing (Tabara et al., 1999; 2002).

DISCUSSION

Here, we show that piRNA and environmental RNAi pathways converge on a common germline nuclear RNAi/chromatin pathway. This pathway can induce stable, multigenerational inheritance. Previous work found evidence for inheritance of small RNAs, chromatin, or both in related transgenerational inheritance paradigms (Burton et al., 2011; Rechavi et al., 2011; Gu et al., 2012). However, here we demonstrate that both small RNA and chromatin factors are essential for multigenerational inheritance and do not act redundantly (Table 1 and Figures 2, 3, S3). We also show that small RNA biogenesis occurs upstream of nuclear RNAi and chromatin factors (Figures 3). Recent work has proposed that somatic nuclear RNAi acts at the level of transcriptional elongation

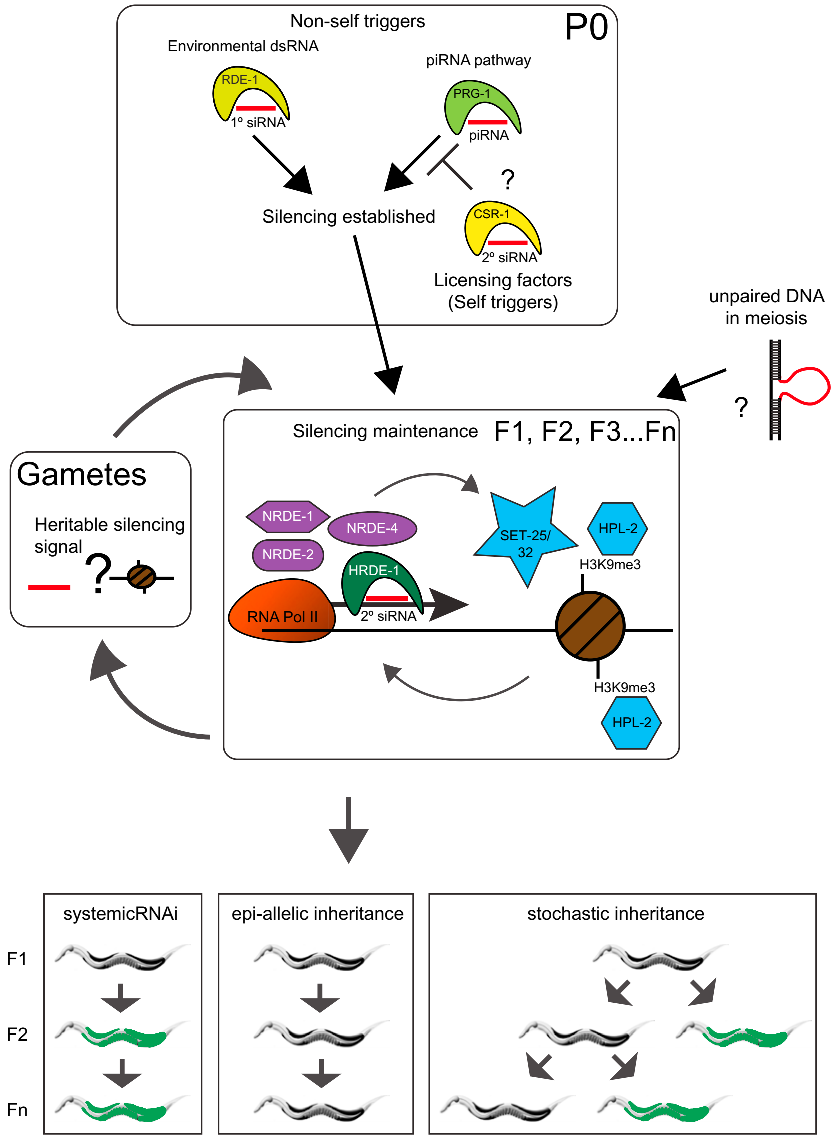

Figure 6. A Model of Transgenerational Silencing in the Germline of *C. elegans*

Triggers such as environmental RNAi and endogenous piRNAs lead to the establishment of a nuclear RNAi/chromatin pathway. Maintenance of silencing requires nuclear RNAi factors, including the germline-specific nuclear Argonaute HRDE-1/WAGO-9 and chromatin proteins such as the HP1 ortholog HPL-2 and the putative histone methyltransferases SET-25 and SET-32. Silencing can be maintained into the F1 for multiple generations (F1–F5) or can become epi-allelic with multigenerational, nonstochastic inheritance. Silencing might be suppressed by a germline licensing pathway that recognizes bona fide germline transcripts (CSR-1 22G-RNA pathway) or might be enforced through the recognition of unpaired DNA during meiosis.

(Guang et al., 2010). These observations opened the possibility that chromatin changes observed in transgenerational inheritance paradigms (Gu et al., 2012) might simply be correlative without being functional in silencing. However, our data show that chromatin factors, such as HPL-2 and SET-25/32, are required (Table 1 and Figures 3 and S3). SET-25/32 are putative histone H3K9me3 methyltransferases. This histone modification, a hallmark of silenced chromatin, has been correlated with small RNA-mediated transgene silencing (Shirayama et al., 2012 [this issue of *Cell*]; Gu et al., 2012) and is enriched on the Hrde sensor reported here (data not shown). In addition, multigenerational silencing of transgenes is promoted by HPL-2 and SET domain proteins (Shirayama et al., 2012). Though this related study did not examine the requirement for SET-25 or SET-32, it did find that MES-4, a histone H3K36 methyltransferase that participates in silencing of the X chromosome (Bender et al., 2006; Rechtsteiner et al., 2010), is also required for multigenerational inheritance. These observations suggest that the chromatin states involved in multigenerational silencing might be complex and could include a hierarchy, which merits further investigation. We have summarized a model of our current understanding of this pathway in Figure 6.

Multicopy versus Single-Copy Transgenes

Multicopy transgenes, intra- or extrachromosomal, are generally efficiently silenced in the germline of *C. elegans* (Kelly et al., 1997). This has been interpreted as an example of the RNAi machinery distinguishing self from nonself (Vastenhouw and Plasterk, 2004). In this model, repetitive DNA such as endogenous transposable elements or multicopy transgenes would give rise to dsRNA that is processed by Dicer to generate siRNA triggers to induce silencing. As the pathways silencing multicopy transgenes and transposable elements share common factors, this phenomenon is of biological interest. However, it has also been a major technical roadblock for researchers studying germ cell biology who rely on reproducible transgene expression in the germline. The advent of MosSCI technology to produce single-copy transgenes has the promise to overcome this problem (Frøkjaer-Jensen et al., 2008, 2012). Interestingly, we and others observed that, in some cases, individual transgenes remain silenced even when present as single, intrachromosomal entities (N.J.L. and E.A.M., unpublished data) (Frøkjaer-Jensen et al., 2008). Indeed, an accompanying paper reports a collection of MosSCI transgenes that remain silenced (Shirayama et al., 2012). Generating MosSCI transgenes in animals in which germline nuclear RNAi pathways are impaired, such as *mut-7*, or "curing" silenced transgenes by outcrossing first to germline RNAi mutants strains and then back to wild-type often results in loss of transgene silencing (N.J.L., A.S., and E.A.M., unpublished data). These results suggest that the original RNAi model of

multicopy transgene silencing needs to be revised. Indeed, there appears to be no requirement for dsRNA intermediates in the silencing phenomena reported here either, as factors required for dsRNA-induced RNAi in the germline such as RSD-2 and RSD-6 (Figure 5 and Table 1) or RDE-1 and RDE-4 (Shirayama et al., 2012) are dispensable for single-copy transgene silencing.

Self versus Nonself

How does *C. elegans* detect single-copy transgenes and target them for silencing in the germline, or how does the animal distinguish self from nonself? The answer might lie in a combination of three factors: scanning germline gene expression by the piRNA pathway (nonself RNA recognition), licensing of germline transcripts (self RNA recognition), and unpaired genomic DNA in meiosis. Based on this and related work, we propose that the piRNA pathway can detect transgenes as sources of foreign RNA (nonself) and initiates targeted silencing of these transgenes (Bagijn et al., 2012; Lee et al., 2012 [this issue of *Cell*]). The piRNA pathway is perfectly suited for this task, as it provides a diverse and large set (~15,000) of small RNA triggers that are mismatch tolerant but do not depend on dsRNA generation (Ruby et al., 2006; Batista et al., 2008; Das et al., 2008; Wang and Reinke, 2008; Bagijn et al., 2012). Furthermore, endogenous germline transcripts are generally depleted in piRNA target sites (Bagijn et al., 2012). In addition, germline licensing pathways might act in the opposite manner to protect bona fide germline transcripts. A recent study reported such a phenomenon in mutants of the *fem-1* locus in *C. elegans* (Johnson and Spence, 2011). In this case, maternal transcripts were required to overcome silencing of an endogenous locus in a mutant background. Furthermore, the germline Argonaute CSR-1 associates with secondary siRNAs that map to many germline-expressed genes without inducing silencing (Claycomb et al., 2009). Interestingly, CSR-1-bound 22G RNAs appear to match abundantly to single-copy transgenes that evade silencing (Shirayama et al., 2012). Taken together, a balance of nonself recognition by the PRG-1/piRNA pathway and self recognition by licensing factors such as the CSR-1 pathway might determine the outcome of gene expression in the germline. This model helps to explain the apparent discrepancy between the facultative multigenerational inheritance that we observe here in our piRNA sensor and the obligatory multigenerational inheritance observed for a related piRNA sensor in a parallel study (Lee et al., 2012; Shirayama et al., 2012). Differences in the composition of the sensors, e.g., the inclusion of the coding region of the *his-58* gene in our sensor, might tip the PRG-1/CSR-1 pathway balance. However, this model fails to explain the ability of our piRNA sensor to be silenced or active depending on its multigenerational ancestry. In our crosses, the piRNA sensor became stably silenced when present in a heterozygous state for several generations (Figures 5 and S5). We propose that unpaired chromatin that has been subjected to silencing by a piRNA trigger can be subjected to an additional layer of silencing during meiosis that then makes the original piRNA trigger dispensable (Figure 6). Unpaired DNA silencing responses have been observed in *C. elegans* in the case of the unpaired X chromosome (Kelly et al., 2002; Bean et al., 2004) and for a mutant *fem-1* locus (Johnson and Spence, 2011) and has also been found in other organisms (Hynes and Todd, 2003; Lee, 2005; Matzke and Birchler, 2005). Establishment of heritable silent chromosome domains that can be robustly maintained in the absence of the original piRNA trigger could be relevant to populations in which sources of piRNAs are polymorphic and may evolve rapidly in response to novel transposons or retroviruses.

Related Phenomena in Other Phyla

The core molecular pathway described here is reminiscent of related (co-) transcriptional pathways both in yeasts and plants (Moazed, 2009). Many yeasts and all plants and nematodes share key factors, such as the RNA-dependent RNA polymerases involved in secondary siRNA generation. Though transgenerational phenomena have been reported in many animals, including humans (Hitchins et al., 2007), this class of polymerases and secondary siRNAs appears to be absent in *Drosophila* and vertebrates. However, it is interesting to note that *Drosophila* and vertebrates have a more complex piRNA system that includes an amplification loop termed “ping-pong,” which could function in a manner analogous to secondary siRNA pathways (Brennecke et al., 2007; Gunawardane et al., 2007). Despite differences in details of piRNA and secondary siRNA systems, common downstream silencing mechanisms may exist.

One, Few, or Many Generations?

Transgenerational phenomena have been observed over one or multiple generations (Grishok et al., 2000; Vastenhouw et al., 2006; Alcazar et al., 2008; Burton et al., 2011; Rechavi et al., 2011; Gu et al., 2012). In some cases, inheritance is stochastic; in others, Mendelian. Here, we report that piRNAs can trigger silencing that lasts for more than 20 generations (Figures 5A, 5B, and S5). Although maintenance of this memory is observed in 100% of offspring, establishment of strong piRNA-independent memory is not obligatory (Figure S5) and only occurs if a silent locus is heterozygous for several generations. This is reminiscent of ubiquitous yet stochastic inactivation of repetitive germline transgenes in many organisms, including *C. elegans*. Our study of transgenes targeted by an endogenous piRNA may recapitulate the fate of transposons that are transmitted in rare horizontal transfer events, in which a single transposon insertion could be subjected to dual layers of silencing, as the locus would likely remain heterozygous for a number of generations before potentially becoming fixed. It will be of great interest to identify the factor(s) that determines these distinct states of silencing.

EXPERIMENTAL PROCEDURES

Genetics

C. elegans were grown under standard conditions at 20°C unless otherwise indicated. The food source used was *E. coli* strain HB101 (*Caenorhabditis* Genetics Center, University of Minnesota, Twin Cities, MN, USA). Bleaching followed by starvation-induced L1 arrest was used to generate synchronized cultures. The wild-type strain was var. Bristol N2 (Brenner, 1974). All strains used are listed in Table S1. For details about genetic crosses, see Supplemental Information.

Transgenics

To generate transgenic animals, germline transformation was performed as described (Mello and Fire, 1995). Injection mixes contained 2–20 ng/μl of MosSCI plasmid and 5–10 ng/μl of marker plasmid DNA (see Supplemental Information for details). Single-copy transgenes were generated by transposase-mediated integration (MosSCI), as described (Frøkjaer-Jensen et al., 2008, 2012).

COPAS Biosort Analysis

A COPAS Biosort instrument (Union Biometrica, Holliston, MA, USA) was used to simultaneously measure length (time of flight), absorbance (extinction), and fluorescence. Data handling and analysis were performed using FlowJo (Tree Star, Inc.) and R.

ACCESSION NUMBERS

The sequence data reported in this paper have been deposited in the Gene Expression Omnibus (GEO) database, http://www.ncbi.nlm.nih.gov/geo, with accession numbers GSE37433 and GSE38813.

SUPPLEMENTAL INFORMATION

Supplemental Information includes Extended Experimental Procedures, five figures, and one table and can be found with this article online at http://dx.doi.org/10.1016/j.cell.2012.06.018.

ACKNOWLEDGMENTS

We thank Sylviane Moss for excellent laboratory management and help with genetic crosses and high-throughput sequencing. We thank Craig Mello and Scott Kennedy for sharing unpublished information. We thank Andrew Bannister and Anne Ferguson-Smith for helpful comments on the manuscript. This work was supported by Cancer Research UK, the Wellcome Trust, and an ERC Starting Grant to E.A.M. and by NIH grant GM083048 to S.A.

Received: June 11, 2012
Revised: June 18, 2012
Accepted: June 18, 2012
Published online: June 25, 2012

REFERENCES

Alcazar, R.M., Lin, R., and Fire, A.Z. (2008). Transmission dynamics of heritable silencing induced by double-stranded RNA in Caenorhabditis elegans. Genetics *180*, 1275–1288.

Alder, M.N., Dames, S., Gaudet, J., and Mango, S.E. (2003). Gene silencing in Caenorhabditis elegans by transitive RNA interference. RNA *9*, 25–32.

Bagijn, M.P., Goldstein, L.D., Sapetschnig, A., Weick, E.M., Bouasker, S., Lehrbach, N.J., Simard, M.J., and Miska, E.A. (2012). Function, targets, and evolution of Caenorhabditis elegans piRNAs. Science. Published online June 14, 2012. http://dx.doi.org/10.1126/science.1220952.

Bateson, W. (1919). Dr. Kammerer's Testimony to the Inheritance of Acquired Characters. Nature *103*, 344–345.

Batista, P.J., Ruby, J.G., Claycomb, J.M., Chiang, R., Fahlgren, N., Kasschau, K.D., Chaves, D.A., Gu, W., Vasale, J.J., Duan, S., et al. (2008). PRG-1 and 21U-RNAs interact to form the piRNA complex required for fertility in C. elegans. Mol. Cell *31*, 67–78.

Bean, C.J., Schaner, C.E., and Kelly, W.G. (2004). Meiotic pairing and imprinted X chromatin assembly in Caenorhabditis elegans. Nat. Genet. *36*, 100–105.

Bender, L.B., Suh, J., Carroll, C.R., Fong, Y., Fingerman, I.M., Briggs, S.D., Cao, R., Zhang, Y., Reinke, V., and Strome, S. (2006). MES-4: an autosome-associated histone methyltransferase that participates in silencing the X chromosomes in the C. elegans germ line. Development *133*, 3907–3917.

Brennecke, J., Aravin, A.A., Stark, A., Dus, M., Kellis, M., Sachidanandam, R., and Hannon, G.J. (2007). Discrete small RNA-generating loci as master regulators of transposon activity in Drosophila. Cell *128*, 1089–1103.

Brenner, S. (1974). The genetics of Caenorhabditis elegans. Genetics *77*, 71–94.

Burton, N.O., Burkhart, K.B., and Kennedy, S. (2011). Nuclear RNAi maintains heritable gene silencing in Caenorhabditis elegans. Proc. Natl. Acad. Sci. USA *108*, 19683–19688.

Bühler, M., Verdel, A., and Moazed, D. (2006). Tethering RITS to a nascent transcript initiates RNAi- and heterochromatin-dependent gene silencing. Cell *125*, 873–886.

Carmell, M.A., Xuan, Z., Zhang, M.Q., and Hannon, G.J. (2002). The Argonaute family: tentacles that reach into RNAi, developmental control, stem cell maintenance, and tumorigenesis. Genes Dev. *16*, 2733–2742.

Chandler, V.L. (2010). Paramutation's properties and puzzles. Science *330*, 628–629.

Claycomb, J.M., Batista, P.J., Pang, K.M., Gu, W., Vasale, J.J., van Wolfswinkel, J.C., Chaves, D.A., Shirayama, M., Mitani, S., Ketting, R.F., et al. (2009). The Argonaute CSR-1 and its 22G-RNA cofactors are required for holocentric chromosome segregation. Cell *139*, 123–134.

Das, P.P., Bagijn, M.P., Goldstein, L.D., Woolford, J.R., Lehrbach, N.J., Sapetschnig, A., Buhecha, H.R., Gilchrist, M.J., Howe, K.L., Stark, R., et al. (2008). Piwi and piRNAs act upstream of an endogenous siRNA pathway to suppress Tc3 transposon mobility in the Caenorhabditis elegans germline. Mol. Cell *31*, 79–90.

Daxinger, L., and Whitelaw, E. (2012). Understanding transgenerational epigenetic inheritance via the gametes in mammals. Nat. Rev. Genet. *13*, 153–162.

Fire, A., Xu, S., Montgomery, M.K., Kostas, S.A., Driver, S.E., and Mello, C.C. (1998). Potent and specific genetic interference by double-stranded RNA in Caenorhabditis elegans. Nature *391*, 806–811.

Frøkjaer-Jensen, C., Davis, M.W., Hopkins, C.E., Newman, B.J., Thummel, J.M., Olesen, S.-P., Grunnet, M., and Jorgensen, E.M. (2008). Single-copy insertion of transgenes in Caenorhabditis elegans. Nat. Genet. *40*, 1375–1383.

Frøkjær-Jensen, C., Davis, M.W., Ailion, M., and Jorgensen, E.M. (2012). Improved Mos1-mediated transgenesis in C. elegans. Nat. Methods *9*, 117–118.

Greer, E.L., Maures, T.J., Ucar, D., Hauswirth, A.G., Mancini, E., Lim, J.P., Benayoun, B.A., Shi, Y., and Brunet, A. (2011). Transgenerational epigenetic inheritance of longevity in Caenorhabditis elegans. Nature *479*, 365–371.

Grishok, A., Tabara, H., and Mello, C.C. (2000). Genetic requirements for inheritance of RNAi in C. elegans. Science *287*, 2494–2497.

Gu, W., Shirayama, M., Conte, D., Jr., Vasale, J., Batista, P.J., Claycomb, J.M., Moresco, J.J., Youngman, E.M., Keys, J., Stoltz, M.J., et al. (2009). Distinct argonaute-mediated 22G-RNA pathways direct genome surveillance in the C. elegans germline. Mol. Cell *36*, 231–244.

Gu, S.G., Pak, J., Guang, S., Maniar, J.M., Kennedy, S., and Fire, A. (2012). Amplification of siRNA in Caenorhabditis elegans generates a transgenerational sequence-targeted histone H3 lysine 9 methylation footprint. Nat. Genet. *44*, 157–164.

Guang, S., Bochner, A.F., Burkhart, K.B., Burton, N., Pavelec, D.M., and Kennedy, S. (2010). Small regulatory RNAs inhibit RNA polymerase II during the elongation phase of transcription. Nature *465*, 1097–1101.

Gunawardane, L.S., Saito, K., Nishida, K.M., Miyoshi, K., Kawamura, Y., Nagami, T., Siomi, H., and Siomi, M.C. (2007). A slicer-mediated mechanism for repeat-associated siRNA 5′ end formation in Drosophila. Science *315*, 1587–1590.

Haig, D. (2006). Weismann Rules! OK? Epigenetics and the Lamarckian temptation. Biol. Philos. *22*, 415–428.

Han, W., Sundaram, P., Kenjale, H., Grantham, J., and Timmons, L. (2008). The Caenorhabditis elegans rsd-2 and rsd-6 genes are required for chromosome functions during exposure to unfavorable environments. Genetics *178*, 1875–1893.

Hitchins, M.P., Wong, J.J.-L., Suthers, G., Suter, C.M., Martin, D.I.K., Hawkins, N.J., and Ward, R.L. (2007). Inheritance of a cancer-associated MLH1 germline epimutation. N. Engl. J. Med. *356*, 697–705.

Hynes, M.J., and Todd, R.B. (2003). Detection of unpaired DNA at meiosis results in RNA-mediated silencing. Bioessays *25*, 99–103.

Johnson, C.L., and Spence, A.M. (2011). Epigenetic licensing of germline gene expression by maternal RNA in C. elegans. Science *333*, 1311–1314.

Jose, A.M., Garcia, G.A., and Hunter, C.P. (2011). Two classes of silencing RNAs move between Caenorhabditis elegans tissues. Nat. Struct. Mol. Biol. *18*, 1184–1188.

Katz, D.J., Edwards, T.M., Reinke, V., and Kelly, W.G. (2009). A C. elegans LSD1 demethylase contributes to germline immortality by reprogramming epigenetic memory. Cell *137*, 308–320.

Kelly, W.G., Xu, S., Montgomery, M.K., and Fire, A. (1997). Distinct requirements for somatic and germline expression of a generally expressed Caenorhabditis elegans gene. Genetics *146*, 227–238.

Kelly, W.G., Schaner, C.E., Dernburg, A.F., Lee, M.-H., Kim, S.K., Villeneuve, A.M., and Reinke, V. (2002). X-chromosome silencing in the germline of C. elegans. Development *129*, 479–492.

Lee, J.T. (2005). Sex chromosome inactivation: the importance of pairing. Curr. Biol. *15*, R249–R252.

Lee, H.-C., Gu, W., Shirayama, M., Youngman, E., Conte, D., Jr., and Mello, C.C. (2012). *C. elegans* piRNAs mediate the genome-wide surveillance of germline transcripts. Cell *150*, this issue, 78–87.

Malone, C.D., and Hannon, G.J. (2009). Small RNAs as guardians of the genome. Cell *136*, 656–668.

Matzke, M.A., and Birchler, J.A. (2005). RNAi-mediated pathways in the nucleus. Nat. Rev. Genet. *6*, 24–35.

Mello, C., and Fire, A. (1995). DNA transformation. Methods Cell Biol. *48*, 451–482.

Merritt, C., Rasoloson, D., Ko, D., and Seydoux, G. (2008). 3′ UTRs are the primary regulators of gene expression in the C. elegans germline. Curr. Biol. *18*, 1476–1482.

Moazed, D. (2009). Small RNAs in transcriptional gene silencing and genome defence. Nature *457*, 413–420.

Pak, J., and Fire, A. (2007). Distinct populations of primary and secondary effectors during RNAi in C. elegans. Science *315*, 241–244.

Rechavi, O., Minevich, G., and Hobert, O. (2011). Transgenerational inheritance of an acquired small RNA-based antiviral response in C. elegans. Cell *147*, 1248–1256.

Rechtsteiner, A., Ercan, S., Takasaki, T., Phippen, T.M., Egelhofer, T.A., Wang, W., Kimura, H., Lieb, J.D., and Strome, S. (2010). The histone H3K36 methyltransferase MES-4 acts epigenetically to transmit the memory of germline gene expression to progeny. PLoS Genet. *6*, e1001091.

Ruby, J.G., Jan, C., Player, C., Axtell, M.J., Lee, W., Nusbaum, C., Ge, H., and Bartel, D.P. (2006). Large-scale sequencing reveals 21U-RNAs and additional microRNAs and endogenous siRNAs in C. elegans. Cell *127*, 1193–1207.

Sijen, T., Steiner, F.A., Thijssen, K.L., and Plasterk, R.H.A. (2007). Secondary siRNAs result from unprimed RNA synthesis and form a distinct class. Science *315*, 244–247.

Shirayama, M., Seth, M., Lee, H.-C., Gu, W., Ishidate, T., Conte, D., Jr., and Mello, C.C. (2012). piRNAs initiate an epigenetic memory of nonself RNA in the C. elegans germline. Cell *150*, this issue, 65–77.

Stam, M., and Mittelsten Scheid, O. (2005). Paramutation: an encounter leaving a lasting impression. Trends Plant Sci. *10*, 283–290.

Suter, C.M., and Martin, D.I. (2010). Paramutation: the tip of an epigenetic iceberg? Trends Genet. *26*, 9–14.

Tabara, H., Sarkissian, M., Kelly, W.G., Fleenor, J., Grishok, A., Timmons, L., Fire, A., and Mello, C.C. (1999). The rde-1 gene, RNA interference, and transposon silencing in C. elegans. Cell *99*, 123–132.

Tabara, H., Yigit, E., Siomi, H., and Mello, C.C. (2002). The dsRNA binding protein RDE-4 interacts with RDE-1, DCR-1, and a DExH-box helicase to direct RNAi in C. elegans. Cell *109*, 861–871.

Tijsterman, M., May, R.C., Simmer, F., Okihara, K.L., and Plasterk, R.H.A. (2004). Genes required for systemic RNA interference in Caenorhabditis elegans. Curr. Biol. *14*, 111–116.

Timmons, L., Court, D.L., and Fire, A. (2001). Ingestion of bacterially expressed dsRNAs can produce specific and potent genetic interference in Caenorhabditis elegans. Gene *263*, 103–112.

Varmuza, S. (2003). Epigenetics and the renaissance of heresy. Genome *46*, 963–967, discussion 968–973.

Vastenhouw, N.L., and Plasterk, R.H.A. (2004). RNAi protects the Caenorhabditis elegans germline against transposition. Trends Genet. *20*, 314–319.

Vastenhouw, N.L., Brunschwig, K., Okihara, K.L., Müller, F., Tijsterman, M., and Plasterk, R.H. (2006). Gene expression: long-term gene silencing by RNAi. Nature *442*, 882.

Wang, G., and Reinke, V. (2008). A C. elegans Piwi, PRG-1, regulates 21U-RNAs during spermatogenesis. Curr. Biol. *18*, 861–867.

Weismann, A. (1891). Essays on heredity (Oxford: Clarendon Press).

Winston, W.M., Molodowitch, C., and Hunter, C.P. (2002). Systemic RNAi in C. elegans requires the putative transmembrane protein SID-1. Science *295*, 2456–2459.

Yigit, E., Batista, P.J., Bei, Y., Pang, K.M., Chen, C.-C.G., Tolia, N.H., Joshua-Tor, L., Mitani, S., Simard, M.J., and Mello, C.C. (2006). Analysis of the C. elegans Argonaute family reveals that distinct Argonautes act sequentially during RNAi. Cell *127*, 747–757.

Zhang, C., Montgomery, T.A., Fischer, S.E., Garcia, S.M., Riedel, C.G., Fahlgren, N., Sullivan, C.M., Carrington, J.C., and Ruvkun, G. (2012). The Caenorhabditis elegans RDE-10/RDE-11 complex regulates RNAi by promoting secondary siRNA amplification. Curr. Biol. *22*, 881–890.

EXTENDED EXPERIMENTAL PROCEDURES

Genetic Crosses

Testing nrde- or prg-1-Mediated Suppression of mjIs144

Marker double mutants *rol-6; dpy-17* or *unc-13; rol-6* were first created to make *prg-1; mjIs144* or *mjIs144*, *nrde-2 or mjIs144*; *nrde-1* strains. To facilitate making strains with *mjIs144*, the *unc-119(ed3)* mutation was first removed from SX1316 by crossing *rol-6 e187* / + males with single SX1316 hermaphrodites, selecting for F1 with Rol F2, singling many non-Rol siblings, selecting for F2 with Rol F3 but no Unc F3, and then selecting against *rol-6* to obtain *mjIs144* homozygotes. *unc-13; mjIs144* and *mjIs144; dpy-17* strains were then created by crossing *rol-6; dpy-17* or *unc-13; rol-6* with N2 wild-type males, crossing F1 *rol-6; dpy-17* / +; + or *unc-13; rol-6* / +; + males with single *mjIs144* hermaphrodites, selecting for F1 that segregated both marker mutations, and then selecting for F2 that lacked *rol-6* and then *unc-13* or *dpy-17* F3. Heterozygous *prg-1* / *unc-13* males stock were maintained by backcrossing heterozygous males with *unc-13* homozygotes. Heterozygous *nrde-1* / *dpy-17* males stock were maintained by backcrossing heterozygous males with *dpy-17* homozygotes. *prg-1; rol-6* strains were crossed with *prg-1* / *unc-13* males, and F1 males were crossed with *unc-13; mjIs144* hermaphrodites, and F1 with both marker mutations were transferred for several generations to obtain strains that lacked Unc or Rol mutations, which were *prg-1; mjIs144* strains. *rol-6; nrde-1* animals were crossed with *nrde-1* / *dpy-17* males, F1 males were crossed with *mjIs144; dpy-17* hermaphrodites, and F1 with both marker mutations were transferred for several generations to obtain strains that lacked Rol or Dpy mutations, which were *mjIs144; nrde-1*. *nrde-2* is tightly linked to the piRNA sensor (*mjIs144*) on chromosome II, so *dpy-10*, piRNA sensor, *unc-4* strains were first created, and then F2 Dpy-non-Unc recombinants from *dpy-10*, *mjIs144*, *unc-4* / +, +, *nrde-2* F1 were singled, allowed to self, and genotyped for the presence of the piRNA sensor. Three of 6 F2 Dpy-non-Unc recombinants possessed the piRNA sensor, and F4 progeny of F3 homozygotes for piRNA sensor, *nrde-2* displayed uniformly GFP. To construct piRNA sensor*; rsd-2* strains, *rsd-2; rol-6* and *unc-24; mjIs144* strains were created using a *rol-6; unc-24* strain. *rol-6; rsd-2* hermaphrodites were then crossed with *rsd-2* males. Male offspring were crossed with *unc-24; mjIs144* hermaphrodites. F1 from this cross were singled. F2 with an unaffected phenotype but with both *rol-6* and *unc-24* siblings were singled. F2 that segregated neither *unc-24*, nor *rol-6* offspring were selected, producing strains homozygous for both *rsd-2* the piRNA sensor. To construct *rsd-6;* piRNA sensor strains, *rsd-6; rol-6* and *dpy-5 unc −55; mjIs144* strains were created using a *dpy-5 unc-55; rol-6* strain. *rsd-6; dpy-10 unc-4* hermaphrodites were then crossed with *rsd-6* males. Male offspring were selected and crossed with *dpy-5 unc-55; mjIs144* hermaphrodites. F1 were singled and those which produced *dpy-5 unc-55* and *dpy-10 unc-4* F2 were selected. Unaffected F2 were singled from these plates. Finally, F2 which segregated neither *dpy-5 unc-55* nor *dpy-10 unc-4* were selected. Independent strains that were homozygous for both *rsd-6* and the piRNA sensor were tested for GFP fluorescence.

Genotyping

SNP Mapping

SNP mapping of mutations was performed as described previously (Bruinsma et al., 2008). Primer sequences are available on request.

Other Genotyping

In order to verify that the *prg-1*; *mjIs144* strains had both the *prg-1 n4357* allele and the *mjIs144* transgene, DNA was extracted from a recently starved plate of each *prg-1*; *mjIs144* strain. PCR was performed using Bam-GFP-FW and Bam-GFP 2 Stop-RV primers to confirm the presence of the transgene. DNA from wild-type worms and *mjIs144* worms were used as controls. The PCR products revealed an approximately 800 bp band indicating the presence of the GFP DNA sequence in the *mjIs114* control as well as all in eight *prg-1*; *mjIs144* strains, but not in the wild-type control. PCR using *n4357* FW and *n4357* RV primers was used to verify the presence of the *prg-1 n4357* allele. DNA from wild-type worms and *prg-1 n4357* worms were used as controls. The PCR products revealed a long band non mutant band for wild-type but only a shorter band for the *n4357* deletion control as well as for all eight *prg-1*; *mjIs144* strains, confirming that the *prg-1*; *mjIs144 n4357* strains were homozygous for *n4357*.

Transgenics

rsd-6 Rescue Transgenes

Rescue constructs were prepared using MultiSite Gateway Three-Fragment Vector Construction Kit (Invitrogen). A PCR product corresponding to the coding region of *rsd-6* was amplified from the fosmid WRM064bA10 (Source Bioscience) and subcloned into pDONR 221. Ubiquitously expressed *dpy-30* promoter and the 3′UTR of *rsd-6* were amplified from N2 wild-type genomic DNA and subcloned into pDONR P4-P1R and pDONR P2R-P3 vectors, respectively. Germline-specific *pgl-3* promoter is a gift from Kyle Wang. Fragments for promoter, gene and 3′UTR were combined into pCFJ150 vector (Frøkjaer-Jensen et al., 2008) including *Caenorhabditis briggsae unc-119*(+) gene. All pDONR subclones were sequenced to confirm lack of mutations, and att recombination sites were sequenced in the final *promoter:gene:3′UTR* constructs prior to microinjection. Primers were following; attB1-RSD6FW, ggggacaagtttgtacaaaaaagcaggctatgaatgaaaaagagctggcggatt; attB2-RSD6RV, ggggaccactttgtacaagaaagctgggtt cagataaagacgtctttgatattc; attB4-dpy30p-FW, ggggacaactttgtatagaaaagttggtctattctcacacctctcc; attB1-dpy30p-RV, ggggactgctt

ttttgtacaaacttgcttggtttttgctcgatttct; attB2-rsd6-3U-FW, ggggacagctttcttgtacaaagtggacttcaaatcatgtttctatctaaa; attB3-rsd6-3U-RV, ggggacaactttgtataataaagttgtctcatgtatattgtttgatgtgaa.

Transgenes were injected into *rsd-6(pk3300) I*, *ttTi5605 II (Mos1); unc-119(ed3)III* and the integrants were obtained using the 'Mos1 excision-induced transgene-instructed gene conversion' method (Frøkjaer-Jensen et al., 2008). Single copy inserts at the *ttTi5605* locus were fully sequenced to confirm a lack of deletions or other mutations prior to use of transgenes for complementation tests. RNAi feeding for *rsd-6* mutants with or without transgenes was performed by placing L4 larvae on P1 RNAi plates, transferring to P2 RNAi plates at 24 hr, removing adults from P2 plates at 48 hr, and scoring plates for unhatched embryos 20 hr after adults were removed.

Microscopy

Differential interference contrast (DIC) and fluorescence imaging was performed using standard methods using an AxioImager A1 upright microscope (Zeiss, Jena, Germany). Images were captured using an ORCA-ER digital camera (Hamamatsu, Hamamatsu, Japan) and processed using OpenLabs 4.0 software (Improvision, Coventry, UK). For Figure 5 GFP expression was monitored by mounting adult worms on agar pads and monitoring 10 to 20 worms per strain using a Nikon E800 epifluorescence microscope and a 40X Plan Fluor objective.

qRT-PCR

RNAi inheritance assay was performed as described above. Silenced animals were sorted using the biosorter and used to generate the F2 generation. Silenced F2 worms were sorted and collected into Trizol ®. Control worms were grown alongside under identical conditions. RNA extraction was performed using standard protocols. cDNA was synthesized from 5 μg total RNA using Superscript III reverse transcriptase (Invitrogen) with random hexamers. qRT-PCR was performed using a ABI7300 Real Time PCR system (Applied Biosystems). Primers sequences are as follows: nascent transcript F: TCTGTCAGTGGAGAGGGTGA; nascent transcript R: TTTAAACTTACCCATGGAACAGG; mRNA F: CTACCTGTTCCATGGCCAAC; mRNA R: GGCATGGCACTCTTGAAAAA.

RNAi Inheritance Assay

RNAi bacteria was inoculated into LB Broth containing Ampicillin (50 μg/ml) for 6 hr at 37°C with shaking. Bacterial cultures were then seeded on to NGM plates containing IPTG (1 mM) and Carbenicillin (25 μg/ml) and grown overnight at room temperature. Adult animals were placed on the bacteria and the cultures grown at 20°C for 4 days. GFP fluorescence in the germline and oocytes of adult offspring was assayed using either a large particle biosorter (Union Biometrica) or a fluorescent microscope (Kramer scientific). Ten (biosorter) or one (microscope) silenced adults were selected and placed on HB101 growth plates to produce the next generation. This procedure was repeated for up to five generations.

Hrde EMS Screen

After EMS treatment following standard protocols, synchronized P2 larvae (L1) were placed onto GFP RNAi plates for 3 days at 20°C. Adult RNAi P0 animals (EMS P2s) were individually picked onto standard HB101 NGM plates and grown at 20°C for 4 days. The resultant F1 (EMS P3) animals were screened for GFP phenotype and any plates with a substantial number of GFP expressing animals kept for secondary screening. Secondary screening was performed as described above for the RNAi inheritance assay. Screening was performed in 8 batches with 600 P2s individualized each time.

piRNA Sensor EMS Screen

F2 or F3 offspring of mutagenized worms were sorted using a Copas Biosort Large-Particle Sorter as described in (Bagijn et al.). Worms sorted as 'GFP ON' were re-selected for sensor de-silencing using an FBS10 Fluorescence Microscope System (Kramer Scientific).

Preparation of Genomic DNA for Whole-Genome Sequencing

We used a pellet of approximately 60-100 μl of packed mixed stage worms and froze at −80°C. For extraction of genomic DNA we followed the Quiagen DNeasy Blood and Tissue kit (animal tissue spin protocol) including an RNase treatment step. We eluted DNA in 2x 200 μl buffer and quantified using the QuBit dsDNA BR assay kit (Invitrogen). Prior to library preparation we cleaned 150 ng of genomic DNA using the DNA Clean& Concentrator-5 Kit (Zymo Research Corp.). We eluted in 15 μl pre-heated water and re-measured DNA concentration using the QuBit dsDNA HS DNA assay kit.

Whole-Genome Library Preparation and Sequencing

We prepared libraries from genomic DNA using the EPICENTRE Biotechnologies Nextera DNA Sample Prep Kit. Tagmentation was performed in HMW Buffer and PCR-amplified libraries were purified either using the DNA Clean & Concentrator-5 Kit (Zymo Research Corp.) or Agencourt AMPure XP beads (Beckman Coulter). Library concentration was measured using the QuBit dsDNA HS DNA assay kit, then fragment length and distribution were determined with the Agilent High Sensitivity DNA assay (Agilent Technologies Inc.). Whole-genome sequencing of simplex libraries was performed on either the Genome Analyzer IIx or the High-Seq 2000 Illumina platform. Sequencing data were analyzed as described previously (Bagijn et al.).

Small RNA Library Generation

P0 and F4 Samples

Total RNA was isolated using the mirVana miRNA isolation kit (Ambion). The total RNA samples were separated on denaturing 15% polyacrylamide gels and 18-30 nt length species selected. Following poly-A tailing, small RNAs were treated with tobacco acid pyrophosphatase (Epicenter). Adapters were then ligated to the 5′ phosphate of the RNA. First-strand cDNA synthesis was performed using an oligo(dT)-adaptor primer and the M-MLV reverse transcriptase. The resulting cDNAs were PCR-amplified to about 20-30 ng/μl in 18-20 PCR cycles using a high fidelity DNA polymerase. cDNA were purified using the Macherey & Nagel NucleoSpin Extract II kit. Libraries were sequenced at CRI using an Illumina GA2x instrument.

P0 and F4 Samples

Total RNA was isolated using TRIsure reagent (Bioline). Total RNA was treated with RNA 5′ polyphosphatase (Epicenter). cDNA libraries were then prepared following the TruSeq Small RNA protocol (Illumina). Libraries were sequenced on a MiSeq machine (Illumina).

Computational Analysis of Small RNA High-Throughput Sequencing Data

Data processing and analyses were performed using custom Perl scripts and the statistical programming environment R. Fastq sequence reads with missing bases or barcodes not matching any of the expected sequences were excluded (if applicable). Reads were trimmed by removing 5′ barcodes, 3′ adapters or 3′ As depending on the protocol used for library generation. Inserts with length 18-30 nucleotides were collapsed to unique sequences, retaining the number of reads for each sequence. The *C. elegans* genome (assembly WS190/*ce6*) and refGene annotation were downloaded from the UCSC Genome Browser website (http://genome.ucsc.edu/) (Kent et al., 2002; Fujita et al., 2011). Perfect matches to the reference genome and transgene constructs (if applicable) were identified using bowtie in -v alignment mode, allowing for multiple matches (Langmead et al., 2009).

Antibody Generation and Immunostaining

The anti-HRDE-1/WAGO-9 polyclonal antisera were generated using Genomic Antibody Technology (GAT) by SDIX (Newark, USA). In brief, DNA immunization was done using cDNA encoding amino acids 12 to 161 of HRDE-1/WAGO-9. Polyclonal sera were subsequently affinity-purified and tested for specificity in Western blotting (data not shown) and immunostaining. Immunostainings were performed on dissected gonads. Animals were dissected in 10 mM Tetramisol on microscopy slides. After freeze-cracking, the tissue was fixed in ice-cold Methanol for 20 min and rehydrated by three washes in 1xPBS/0.2% Tween 20 (10 min each, room temperature).

Primary antibodies were added over night at 4°C in 1xPBS/0.2% Tween 20/1% BSA. Slides were washed three times in 1x PBS/0.2% Tween 20 (10 min each, room temperature). Secondary antibodies were added for one hour at 37°C. The secondary antibody mix contained 4′,6-diamidino-2-phenylindole (DAPI) at 100 ng/ml final concentration. Slides were washed three times in 1xPBS/0.2% Tween 20 (10 min each, room temperature) and mounted using Vectashield. We carried out differential interference contrast (DIC) and confocal fluorescence imaging using standard methods and using an Olympus FluoView FV1000 upright microscope using 40x and 63x objective magnification. Images were processed using the FluoView image software (Olympus) and ImageJ (version 1.43u). Antibodies and concentrations used are: rabbit anti-HRDE-1/WAGO-9 1:4000, mouse OIC1D4 (Developmental Studies Hybridoma Bank, University of Iowa) 1:100, mouse anti-α-Tubulin (clone DM1A, SIGMA) 1:10,000, Alexa Fluor 488 anti-rabbit IgG, 1:1000, Alexa Fluor 568 anti-mouse IgG, 1:1000 (both Life Technologies).

SUPPLEMENTAL REFERENCES

Bagijn, M.P., Goldstein, L.D., Sapetschnig, A., Weick, E.M., Bouasker, S., Lehrbach, N.J., Simard, M.J., and Miska, E.A. (2012). Function, targets, and evolution of Caenorhabditis elegans piRNAs. Science. Published online June 14, 2012. http://dx.doi.org/10.1126/science.1220952.

Bruinsma, J.J., Schneider, D.L., Davis, D.E., and Kornfeld, K. (2008). Identification of mutations in Caenorhabditis elegans that cause resistance to high levels of dietary zinc and analysis using a genomewide map of single nucleotide polymorphisms scored by pyrosequencing. Genetics *179*, 811–828.

Frøkjaer-Jensen, C., Davis, M.W., Hopkins, C.E., Newman, B.J., Thummel, J.M., Olesen, S.-P., Grunnet, M., and Jorgensen, E.M. (2008). Single-copy insertion of transgenes in Caenorhabditis elegans. Nat. Genet. *40*, 1375–1383.

Fujita, P.A., Rhead, B., Zweig, A.S., Hinrichs, A.S., Karolchik, D., Cline, M.S., Goldman, M., Barber, G.P., Clawson, H., Coelho, A., et al. (2011). The UCSC Genome Browser database: update 2011. Nucleic Acids Res. *39*, D876–D882.

Kent, W.J., Sugnet, C.W., Furey, T.S., Roskin, K.M., Pringle, T.H., Zahler, A.M., and Haussler, D. (2002). The human genome browser at UCSC. Genome Res. *12*, 996–1006.

Langmead, B., Trapnell, C., Pop, M., and Salzberg, S.L. (2009). Ultrafast and memory-efficient alignment of short DNA sequences to the human genome. Genome Biol. *10*, R25.

Lin, R., Thompson, S., and Priess, J.R. (1995). pop-1 encodes an HMG box protein required for the specification of a mesoderm precursor in early C. elegans embryos. Cell *83*, 599–609.

Watts, J.L., Etemad-Moghadam, B., Guo, S., Boyd, L., Draper, B.W., Mello, C.C., Priess, J.R., and Kemphues, K.J. (1996). par-6, a gene involved in the establishment of asymmetry in early C. elegans embryos, mediates the asymmetric localization of PAR-3. Development *122*, 3133–3140.

Cell

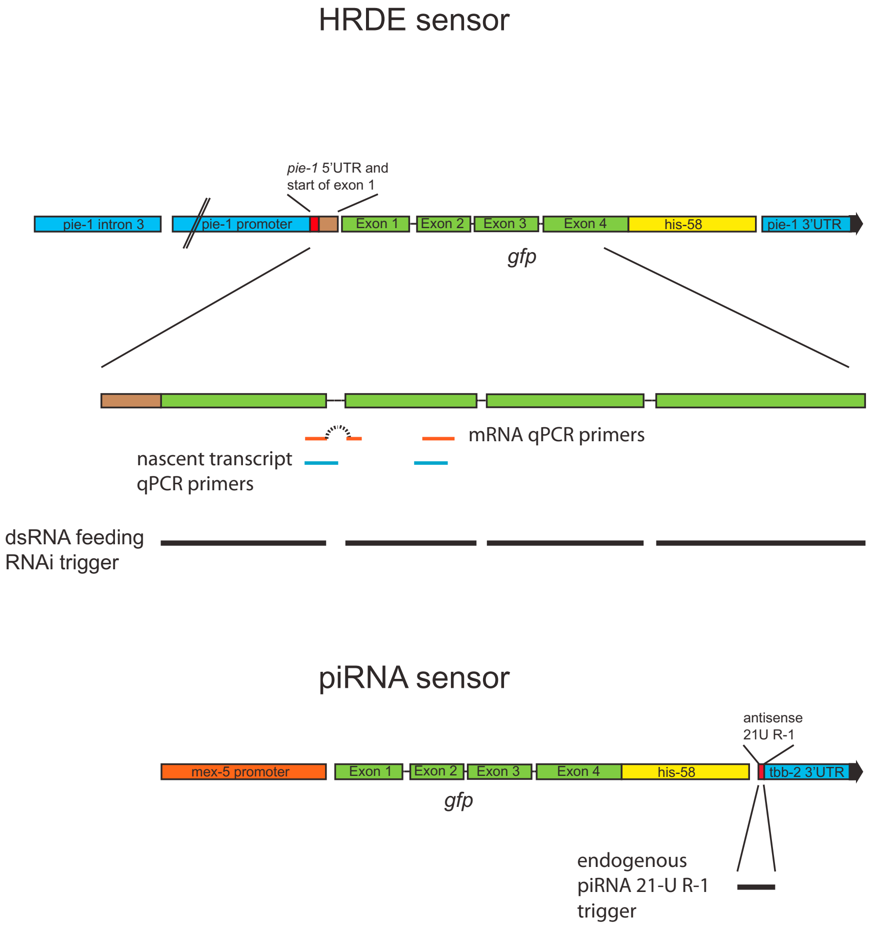

Figure S1. The Hrde Sensor, Related to Figure 1A

Cartoon showing the components of the Hrde sensor transgene used in the transgenerational inheritance assays (top panel) and the piRNA sensor (bottom panel). Also indicated are the locations of the primers used for qRT-PCR analysis, the location of the dsRNA feeding trigger and the location of the piRNA 21U trigger.

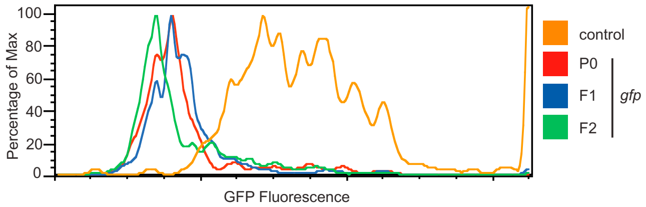

Figure S2. Quantification of GFP Fluorescence in the Hrde Sensor Assay Using Flow Cytometry, Related to Figure 1C
Representative, smoothed histograms showing relative GFP fluorescence of control, GFP RNAi, F1 and F2 wild-type adult animals. Raw data is gated to exclude larval animals, which cannot express the transgene. GFP fluorescence is on an arbitrary scale.

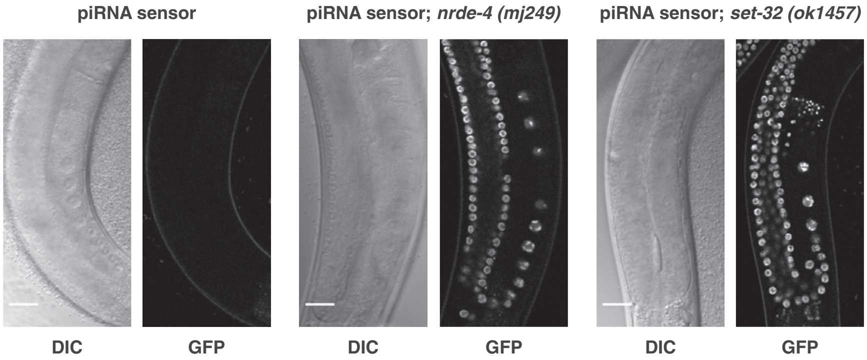

Figure S3. A Nuclear RNAi and Chromatin Factor Are Required for piRNA Sensor Silencing, Related to Figure 3B
DIC and fluorescence microscopy of piRNA sensor germlines in wild-type and indicated mutant genotypes. Scale bars are 20 μm.

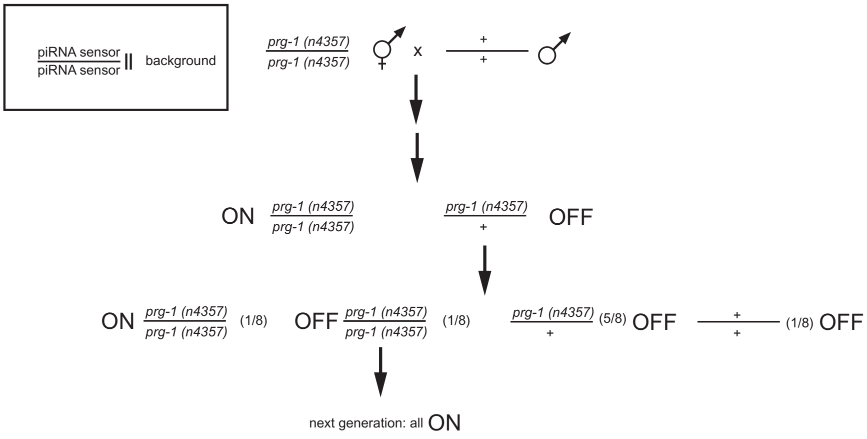

Figure S4. piRNA Sensor Silencing Can be PRG-1 Dependent, Related to Figure 5

Schematic exemplifying a cross where introduction of the piRNA sensor into a *prg-1* mutant background leads to sensor activation as described previously (Bagijn et al., 2012). Subsequent re-introduction of a wild-type *prg-1* allele is not sufficient to induce inheritance of silencing in the *prg-1* mutant offspring.

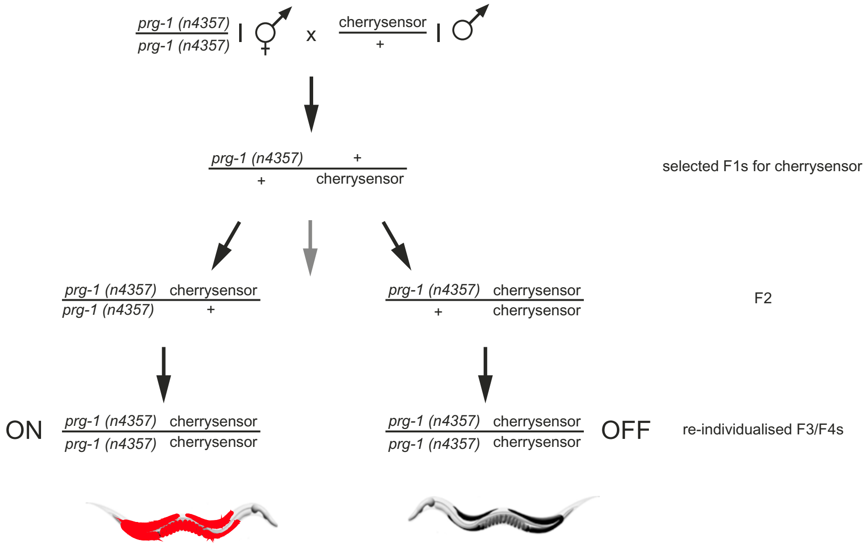

Figure S5. Multigenerational Inheritance of Silencing of the piRNA mCherry Sensor, Related to Figure 5

We crossed *prg-1(n4357)* I mutant hermaphrodites to males carrying one allele of our piRNA mCherry sensor and PCR-selected for mCherry sensor heterozygous F1 offspring. In the F2 generation we used one line each of worms homozygous for one of the desired alleles and heterozygous for the respective other allele and re-individualized F3s to obtain both alleles as homozygotes. In the F3 generation we obtained no double mutants from the *prg-1(n4357)* homozygous F2 but observed mCherry sensor expression in a line heterozygous for the mCherry sensor. Offspring of this F3 was individualized again and we obtained one line mutant for both alleles. This line stably expresses mCherry in the germline. Conversely, we obtained 2 F3 lines of double mutants coming from the mCherry sensor homozygous F2, both of which did not reactivate mCherry expression.

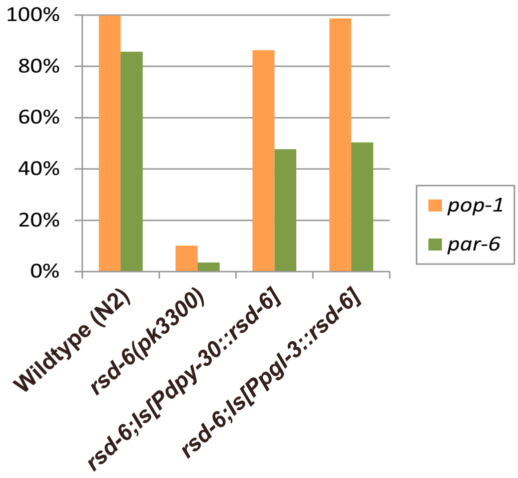

Figure S6. RSD-6 Is Required in Germ Cells for Environmental dsRNA, Related to Figure 5C

Bacteria expressing dsRNA targeting *pop-1* or *par-6*, which are expressed in the maternal germline to promote embryogenesis (Lin et al., 1995; Watts et al., 1996), cause highly penetrant embryonic lethality when fed to wild-type but not to *rsd-6* mutant hermaphrodites. Single copy transgenes that express *rsd-6* ubiquitously (*Pdpy-30::rsd-6*) or in the germline (*Ppgl-3::rsd-6*) both rescue the responses of an *rsd-6* mutation to *pop-1* or *par-6* dsRNA.
